# Deep time dietary convergence evidenced by the stomach contents of a fossil seal from the Southwest Atlantic

**DOI:** 10.1098/rsos.260705

**Published:** 2026-10-21

**Authors:** Ana M. Valenzuela-Toro, Felipe Busker, José Cuitiño, Néstor García, Mónica R. Buono

**Affiliations:** ^1^ Área Científica, Centro de Investigación y Avance de la Paleontología e Historia Natural de Atacama (CIAHN Atacama), Caldera, Región de Atacama, Chile; ^2^ Department of Paleobiology, National Museum of Natural History, Smithsonian Institution, Washington DC, USA; ^3^ Instituto Patagónico de Geología y Paleontología (IPGP), CCT CONICET CENPAT, Puerto Madryn, Chubut, Argentina; ^4^ Centro para el Estudio de Sistemas Marinos, Puerto Madryn, Chubut, Argentina

**Keywords:** pinniped, Phocidae, Phocinae, Monachinae, Miocene, Patagonia, fossilized stomach contents, Southern Hemisphere, marine megafauna

## Abstract

The Neogene fossil record of Patagonia provides important insights into the evolution and ecological diversification of marine predators in the Southern Hemisphere. Among these, *Kawas benegasorum*, an extinct seal from the Late Miocene Puerto Madryn Formation, represents the only record of a putative phocine (northern seals) in the Southern Hemisphere and preserves rare evidence of stomach contents. However, its original description was concise, and lacked detailed information on its dentition and stomach contents, leaving aspects of its morphology, phylogeny and paleoecology unresolved. We re-describe the holotype and only known specimen of *K. benegasorum*, providing the first comprehensive study of its postcranial morphology, new information on its dentition and a detailed examination of its stomach contents. We show that *Kawas* has both monachine- and phocine-like traits, with an overall morphology suggesting affinities with Monachinae. However, phylogenetic analyses yielded conflicting results, recovering *Kawas* either within Phocinae or in an unresolved position within Phocidae. The stomach contents indicate a piscivorous diet with coastal, benthic and demersal prey, broadly comparable to that of extant South American sea lions inhabiting the region. These results document early benthic nearshore foraging in pinnipeds and suggest dietary convergence across geological time in the western South Atlantic by the Late Miocene.

## 1. Introduction

Exceptional preservation in the fossil record offers rare glimpses into the ecology of extinct organisms. Beyond identifying individual prey, fossilized stomach contents can reveal long-term trophic interactions and ecological roles that may reflect broader ecological patterns [1,2]. Such direct evidence is particularly critical for marine megafauna, where foraging behaviours often cannot be inferred from skeletal morphology or geochemical proxies alone. In this context, understanding the feeding preferences and other ecological patterns of marine predators, such as pinnipeds (true seals, fur seals, sea lions and walruses), is key to reconstructing the energy flow and functional structure of ancient marine ecosystems [3].

Pinnipeds are globally distributed and possess a rich fossil record; however, fossilized stomach contents are exceedingly rare [4]. A notable exception is *Kawas benegasorum*, an extinct seal from the Late Miocene Puerto Madryn Formation of Patagonia, Argentina. The holotype and only known specimen of *Kawas* (MPEF-PV 601) consists of an articulated partial skeleton that preserves abundant fish remains within the abdominal region [5]. Despite the significance of this discovery, the original description was brief, did not describe or illustrate the preserved dental remains, and lacked a detailed analysis of the stomach contents, leaving aspects of *Kawas* morphology and paleoecology unresolved.

Beyond its exceptional preservation, *Kawas* has an important place in the evolutionary history of seals [6]. Based on comparative morphology, this species was initially placed within the Phocinae, a clade otherwise restricted to the Northern Hemisphere, making *Kawas* the first and only record of an extinct phocine in the Southern Hemisphere [5]. This classification relied mainly on limb morphology; however, recent studies have documented the occurrence of substantial intra- and interspecific variation in pinniped postcranial morphology, raising questions about the phocine affinity of *Kawas* and its broader phylogenetic relationships [7–10].

At the same time, despite the rich fossil record of marine mammals in Argentina, pinniped remains are rare and fragmentary, a pattern consistent with global trends likely driven by taphonomic, sampling and publishing biases [11]. To date, only four occurrences have been reported, namely *Properiptychus argentinus* from the Paraná Formation [12], *K. benegasorum* [5] and two unidentified Phocidae specimens from the Puerto Madryn Formation ([13]; F Busker, M Buono 2025, personal observation). Therefore, a detailed reassessment of *Kawas*, integrating comparative morphology, phylogenetic analysis and stomach content data, is warranted and provides a critical opportunity to clarify the evolutionary relationships and ecological role of this species, enhancing the understanding of pinniped evolutionary history along the Atlantic coast of South America.

In this work, we re-describe the holotype of *K. benegasorum* from the Puerto Madryn Formation. We provide a detailed description of the postcranial and dental anatomy, and conduct the first comprehensive analysis and illustration of the preserved stomach contents. Using this new anatomical information, we conduct a phylogenetic analysis to test the placement of *Kawas* within Phocidae. By integrating morphological and dietary evidence, we reassess the evolutionary relationships of this enigmatic seal species and reconstruct its foraging behaviour, offering new insights into the structure of Miocene marine food webs and revealing broader patterns of dietary convergence through geological time.

## 2. Material and Methods

### 2.1. Stratigraphic setting and age

The Late Miocene Puerto Madryn Formation consists of a 150 m thick succession of bioturbated fine sandstones, bioturbated mudstones, shell-beds, cross-bedded sandstones and heterolithic beds, accumulated in inner shelf, shore face, tidal channel and tidal flat palaeoenvironments [14–17]. The unit mostly crops out along the coastal cliff of the Valdés Península and surrounding areas in northeastern Patagonia, Argentina (figure 1A,B). The skeleton of *K. benegasorum* comes from bioturbated muddy sandstones at Punta San Román in Valdés Peninsula (figure 1C).

**Figure 1.**
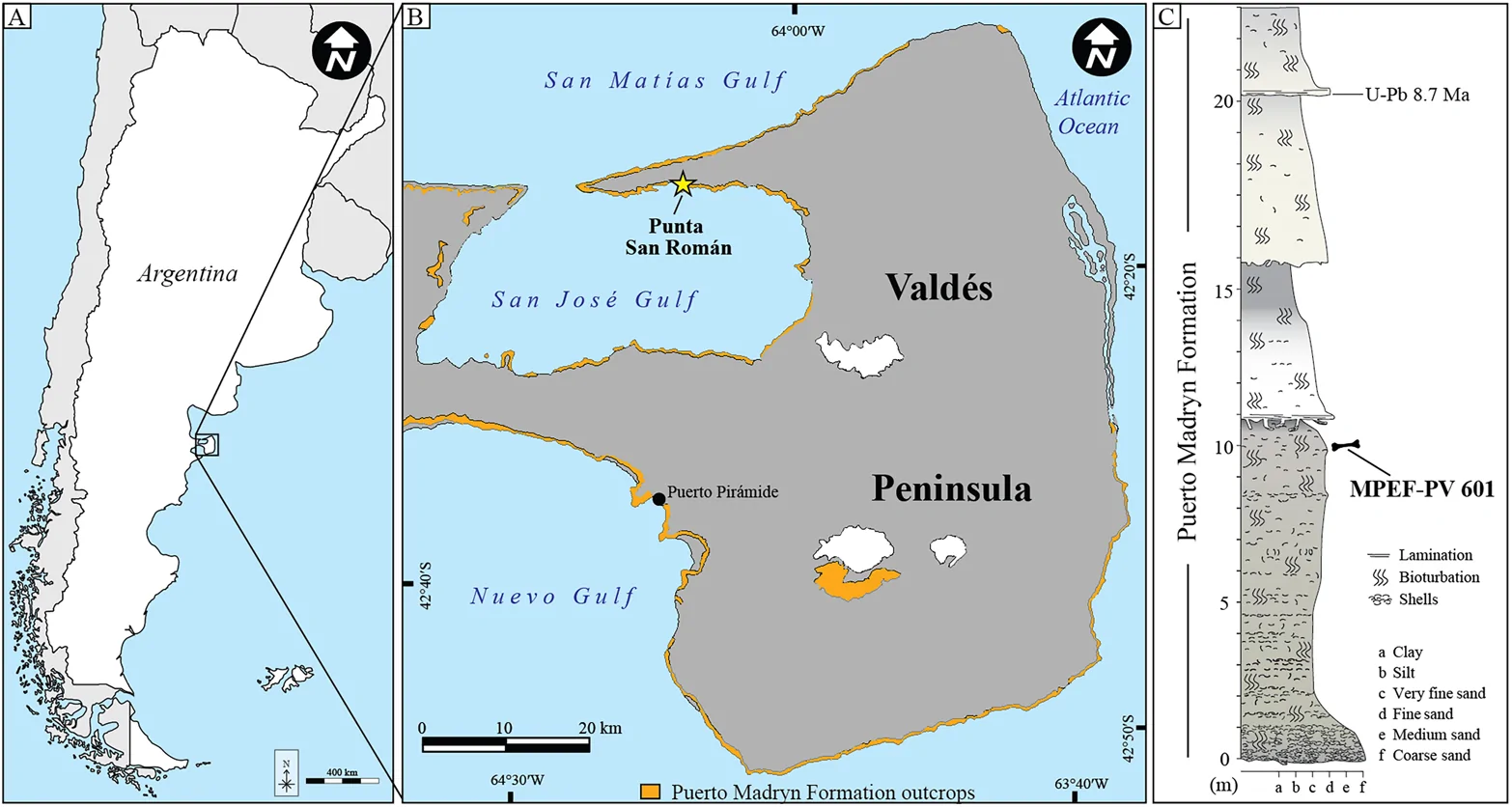
(A–C) Map and stratigraphic column of the Puerto Madryn Formation. Orange areas in (B) indicate outcrops of the Puerto Madryn Formation in the Valdés Peninsula. Colours in (C) are illustrative and represent the sedimentary colourimetry observed in the field for the levels of the formation.

At Punta San Román, the Puerto Madryn Formation is present in small coastal cliffs and wave-cut platforms, forming a succession of about 20 m (figure 1C). In a nearby locality, Farroni *et al.* [18] described a highly fossiliferous succession composed of an alternation of bioturbated sandy, bioclastic and muddy beds, with a variable proportion of volcanic ash, accumulated in the inner shelf to lower shore face environments. Based on available provenance information, MPEF-PV 601 comes from the wave-cut platform at Punta San Román, which we assign to Level 2 of Farroni *et al.* [18] and interpret as inner shelf to lower shore face deposits.

Based on regional correlations, both horizons are approximately correlated, and the same age is assigned to them. They correspond to the lower part of the Puerto Madryn Formation, in levels ascribed to the Transgressive and Maximum Highstand phases by del Río *et al.* [15]. According to ^87^Sr/^86^Sr (Scasso *et al.* [19],[20]) and U-Pb [21] dating, the lower to middle levels of the Puerto Madryn Formation are approximately 12–9 Ma (Tortonian). Near Punta San Román, a tuff horizon located 15 m above the *Kawas* horizon is dated at 8.67 Ma [21]; therefore, we propose that the bearing horizon should be close to 10–9 Ma.

### 2.2. Institutional abbreviations

**CAS MAM**, Ornithology and Mammalogy Collection, California Academy of Sciences, San Francisco, California, USA; **IRSNB**, Institut Royal des Sciences Naturelles de Belgique, Brussels, Belgium; **FFM**, Feldman Family Museum, Kharkiv, Ukraine; **LACM**, Department of Mammalogy and Vertebrate Paleontology Collection, Natural History Museum of Los Angeles County, Los Angeles, California, USA; **MACN**, Museo Argentino de Ciencias Naturales Bernardino Rivadavia, Buenos Aires, Argentina; **MNHN.F.**, Muséum National d'Histoire Naturelle, Paris, France; **MPEF-PV**, Vertebrate Paleontology Collection, Museo Paleontológico Egidio Feruglio, Trelew, Chubut, Argentina; **MSNUP**, Museo di Storia Naturale, Universita di Pisa, Pisa, Italy; **MUSM**, Museo de Historia Natural, Universidad Nacional Mayor de San Marcos, Lima, Peru; **SDNHM**, San Diego Natural History Museum, San Diego, California, USA; **SGO.PV**, Museo Nacional de Historia Natural, Santiago, Chile; and **USNM** and **USNM PAL**, Departments of Vertebrate Zoology and Paleobiology, respectively, National Museum of Natural History, Smithsonian Institution, Washington, D.C., USA.

### 2.3. Anatomical descriptions and comparisons

Following the framework of Rule *et al.* [10], morphological comparisons were restricted to valid phocid taxa, excluding those currently considered as nomina dubia. Linear measurements of *K. benegasorum* were taken by one of the authors (F. Busker) using a manual calliper and were modified from Ericson & Storå [22], Valenzuela-Toro *et al.* [23], Dewaele & de Muizon [8], among others. Comparative fossil material was examined through both direct observation and published descriptions, including *Acrophoca longirostris* (MNHN.F.SAS563; SGO.PV 22110), *Acrophoca* sp. (SGO.PV 22109), Phocidae indet. (USNM 181419), *Australophoca changorum* (USNM 438707 and 438712; SGO.PV 21569 and 22106; MUSM 569/SAS 989), *Hadrokirus martini* (SGO.PV 21108 and 22126; MNHN.F.SAS 1627), *Homiphoca* sp. (USNM 187228), *Leptophoca* (IRSNB 1146-M279 and 1145-M280a), *Magophoca brevirostris* (MNHN.F.PPI276, 269, 276, 279, 280, 282 and 295), *Nanophoca vitulinoides* (IRSNB M2276a, M2276c, M2276e, M2272, M2277, 1063-M242 and 1066-M243b), *Piscophoca pacifica* (MNHN.F.SAS564) and *Pliophoca etrusca* (MSNUP I-13993), as well as additional unidentified fossil Monachinae and Phocinae referenced in the text. Comparative material of extant Phocidae included *Hydrurga leptonyx* (USNM 275208, 270326, 275205, 275208 and 550358), *Leptonychotes weddellii* (USNM 269530, 550359 and 504875), *Lobodon carcinophagus* (USNM 269722), *Neomonachus schauinslandi* (LACM 54438 and 54384; SDNHM 18711; USNM 395999), *Neomonachus tropicalis* (USNM 102536), *Mirounga angustirostris* (USNM A 21890, 260867, 265353, A 20927 and A 21886), *Ommatophoca rossii* (USNM 339989, 275206 and 270321), *Halichoerus grypus* (LACM 84079; CAS MAM 22571; USNM 446405, 446406 and 446408), *Pusa hispida* (LACM 54781; SDNHM 12213), *Phoca vitulina* (USNM 6486 and 250712) and *Pusa sibirica* (CAS MAM 12186; LACM 54781).

### 2.4. Phylogenetic analysis

To analyse the phylogenetic position of *K. benegasorum*, we used a modified version of the total-evidence data matrix of Rule *et al.* [9], only considering the morphological data, which includes 168 morphological characters scored for 42 taxa. Of those taxa, 21 are extinct fossil species, and one represents a species extinct in historical times (*Neomonachus tropicalis*). As in the original file, we keep characters 48, 85, 142 and 159 as ordered.

We modified the coding of five characters for *Kawas* based on our redescription of the holotype. Specifically, we modified character 14 (M1, from unknown to state 1, ‘smaller than P4’); character 16 (elevation of the labial cingulum below the main cusp of post-canine teeth, from state 0, ‘absent,’ to state 1, ‘present’); character 121 (humerus, lateral epicondylar crest, from state 0, ‘well developed,’ to state 1, ‘absent or poorly developed’); character 138 (manus, phalanges, from unknown to state 1, ‘flattened’); and character 152 (femur, neck, from state 0, ‘slender,’ to state 1, ‘short and wide’). Furthermore, we added four additional taxa: *Icaphoca choristodon* [24], *Magophoca brevirostris* [8], *Monachopsis pontica* [25] and *Paratethyphoca libera* [26] (supplementary material, table S1) to the matrix, resulting in a final dataset of 46 taxa, including 25 extinct species. Missing and inapplicable data represent 31.80% of the dataset (29.32% from extinct taxa).

For the phylogenetic analyses, a heuristic parsimony analysis was performed in TNT v.1.6. The analysis consists of 1000 replications of Wagner trees using random addition sequences, followed by the tree bisection and reconnection (TBR) branch-swapping algorithm (holding 10 trees per replicate). The best trees obtained at the end of the replicates were subjected to a final round of TBR branch swapping. We tried different approaches to analyse the data. An equally weighted parsimony was used to minimize the number of postulated evolutionary transformations. We also performed an analysis with implied weighting (K = 3), which has been shown to improve topological accuracy and stability [27,28]. Finally, we performed an analysis in TNT with a molecular constraint to test the position of the different extinct species, setting the relationships of extant taxa. We used as a constraint the same molecular tree from Rule *et al.* [29]. We conducted the same search strategy described above, but enforcing constraints based on the molecular tree. We summarized the information in a strict consensus tree of each analysis. For support values, we conducted a bootstrap analysis in TNT, with 1000 replicates and a 50% cut-off. Also, the retention and consistency indices (RI and CI) were calculated.

## 3. Systematic palaeontology

CARNIVORA Bowdich, 1821

PINNIPEDIA Illiger, 1811
PHOCIDAE Grey, 1821 (*sensu* Berta & Wyss [30])
*KAWAS BENEGASORUM* [5]
(figures 2–8)

**Figure 2.**
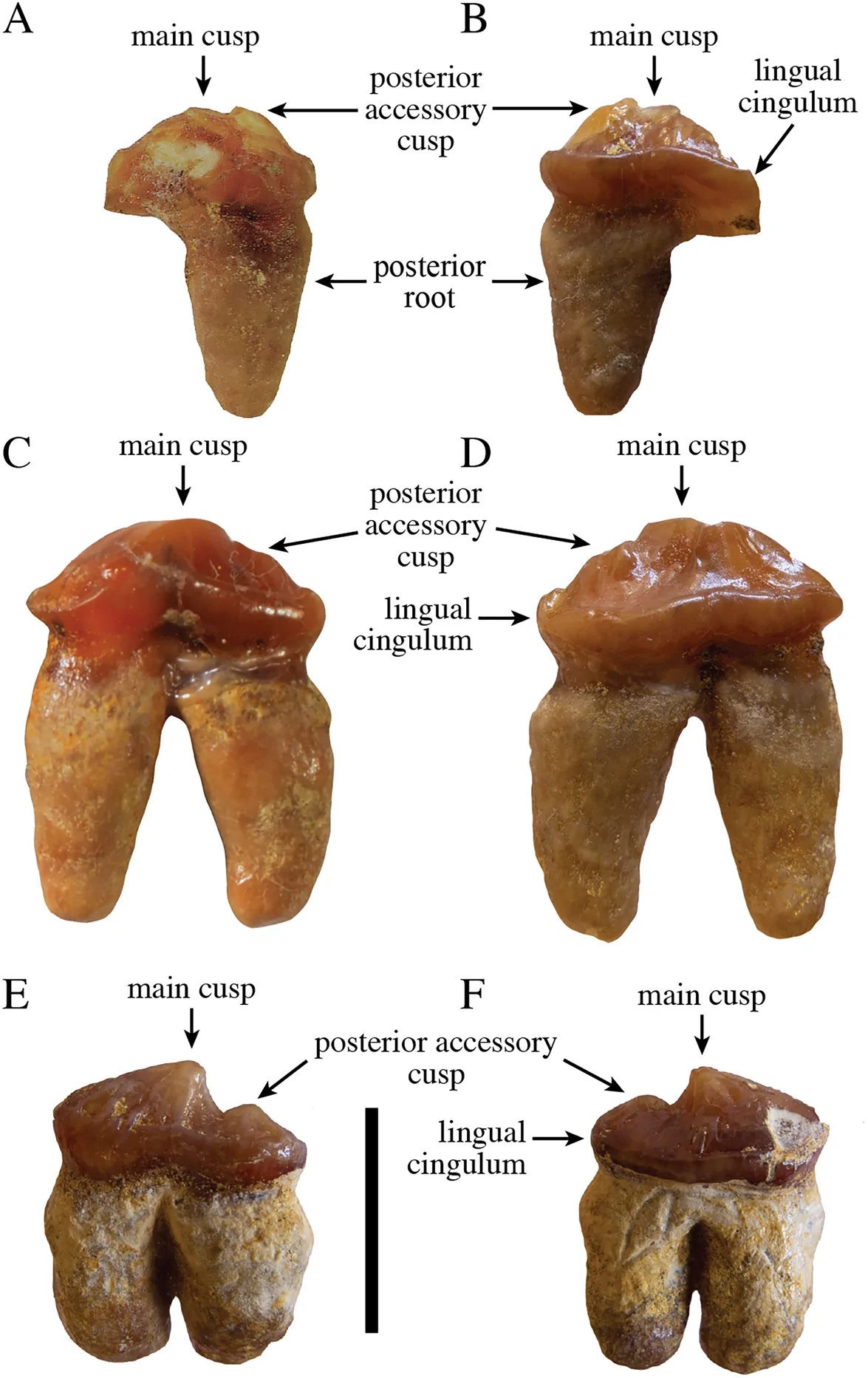
*Kawas benegasorum* (MPEF-PV 601) dentition. Left P3 or P2 in labial (A) and lingual (B) views; P4 in labial (C) and lingual (D) views and M1 in labial (E) and lingual (F) views. Scale bar = 1 cm.

**Figure 3.**
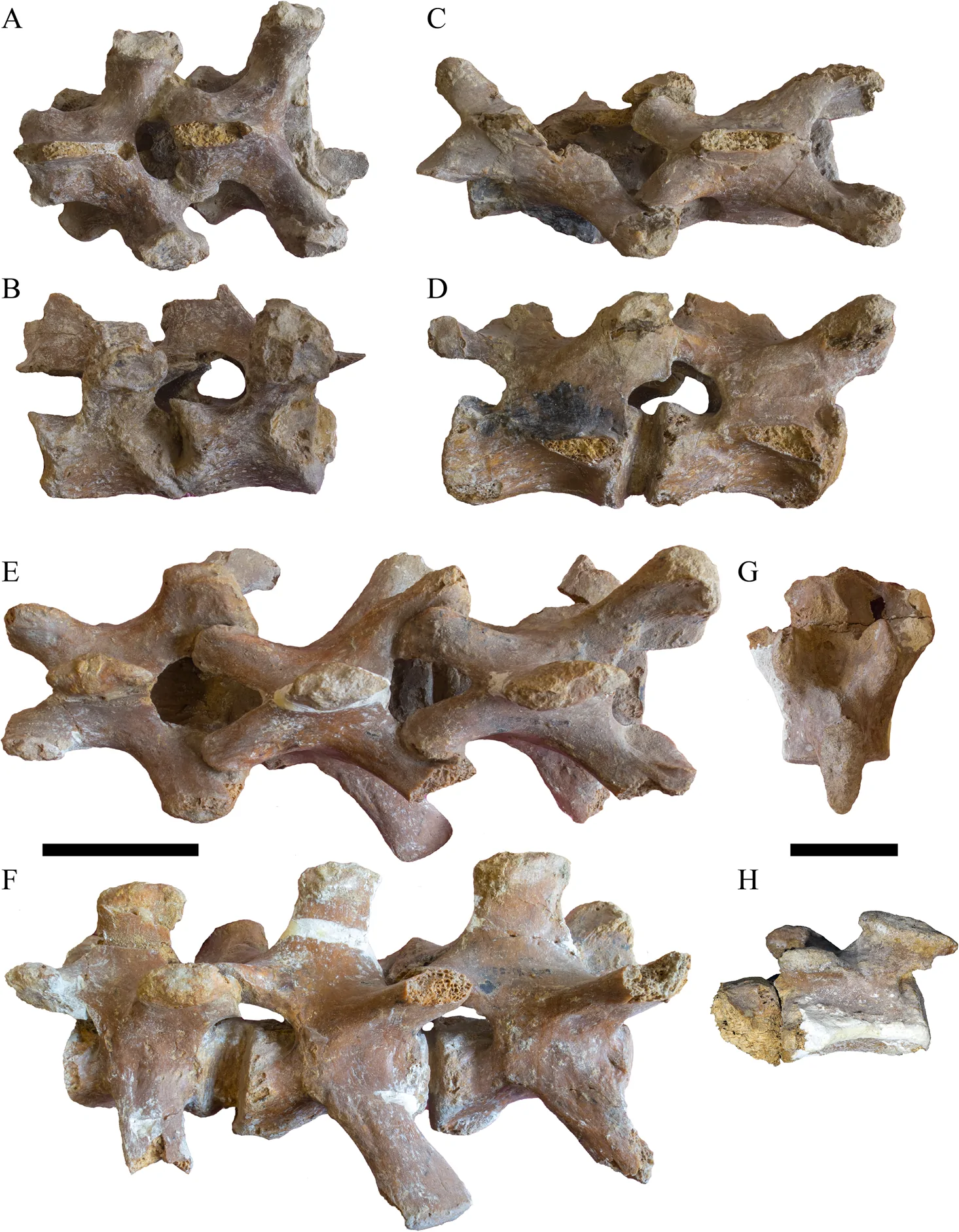
*Kawas benegasorum* (MPEF-PV 601) vertebrae. Selected pre-anticlinal thoracic vertebrae in dorsal (A) and right lateral (B) views. Lumbar vertebrae in dorsal (C, E) and right lateral (D, F) views. Caudal vertebrae (G, H). Scale bar = 5 cm in (A–F), and = 2.5 cm in (G) and (H).

**Figure 4.**
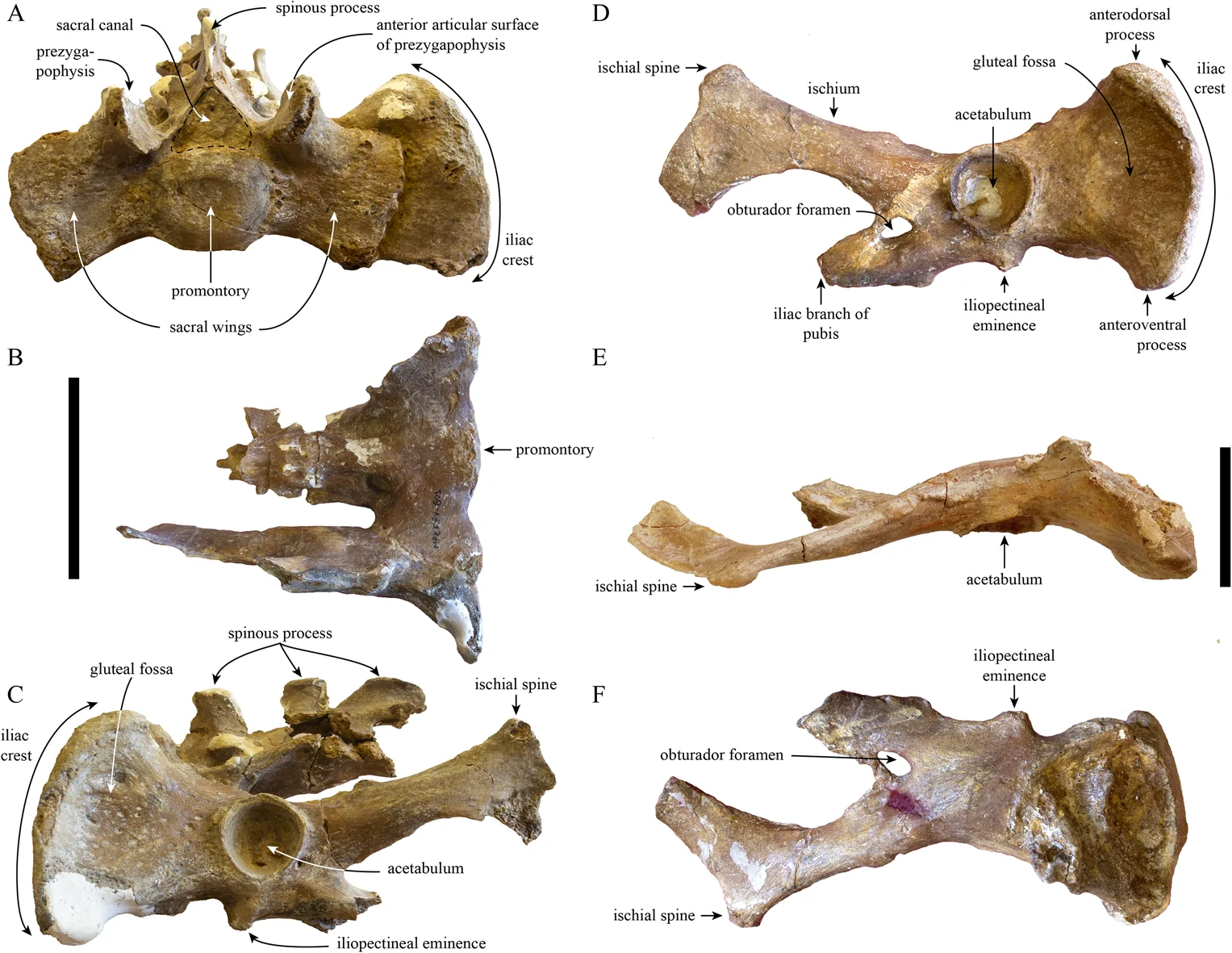
*Kawas benegasorum* (MPEF-PV 601) pelvic bones. Sacrum in proximal (A) and ventral (B) views. Left innominate cemented to the sacrum in lateral (C) view. Right innominate in lateral (D), dorsal (E) and medial (F) views. Scale bar = 10 cm in A–C and = 5 cm in D–F.

**Figure 5.**
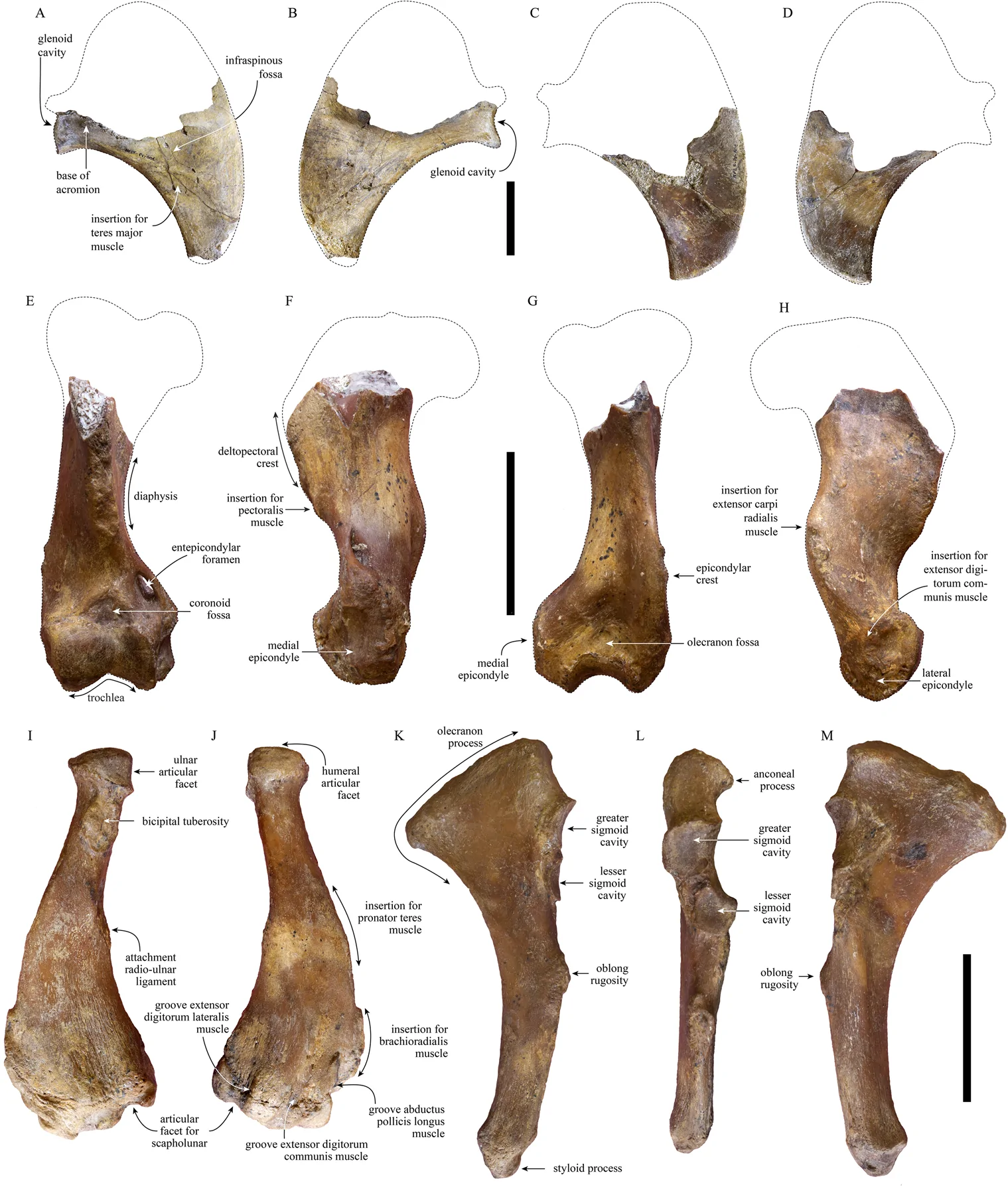
*Kawas benegasorum* (MPEF-PV 601) forelimb bones. Left scapula in lateral (A) and medial (B) views. Right scapula in medial (C) and lateral (D) views. Right humerus in anterior (E), medial (F), posterior (G) and lateral (H) views. Right radius in medial (I) and lateral (J) views. Right ulna in lateral (K), anterior (L) and medial (M) views. Scale bars = 5 cm.

**Figure 6.**
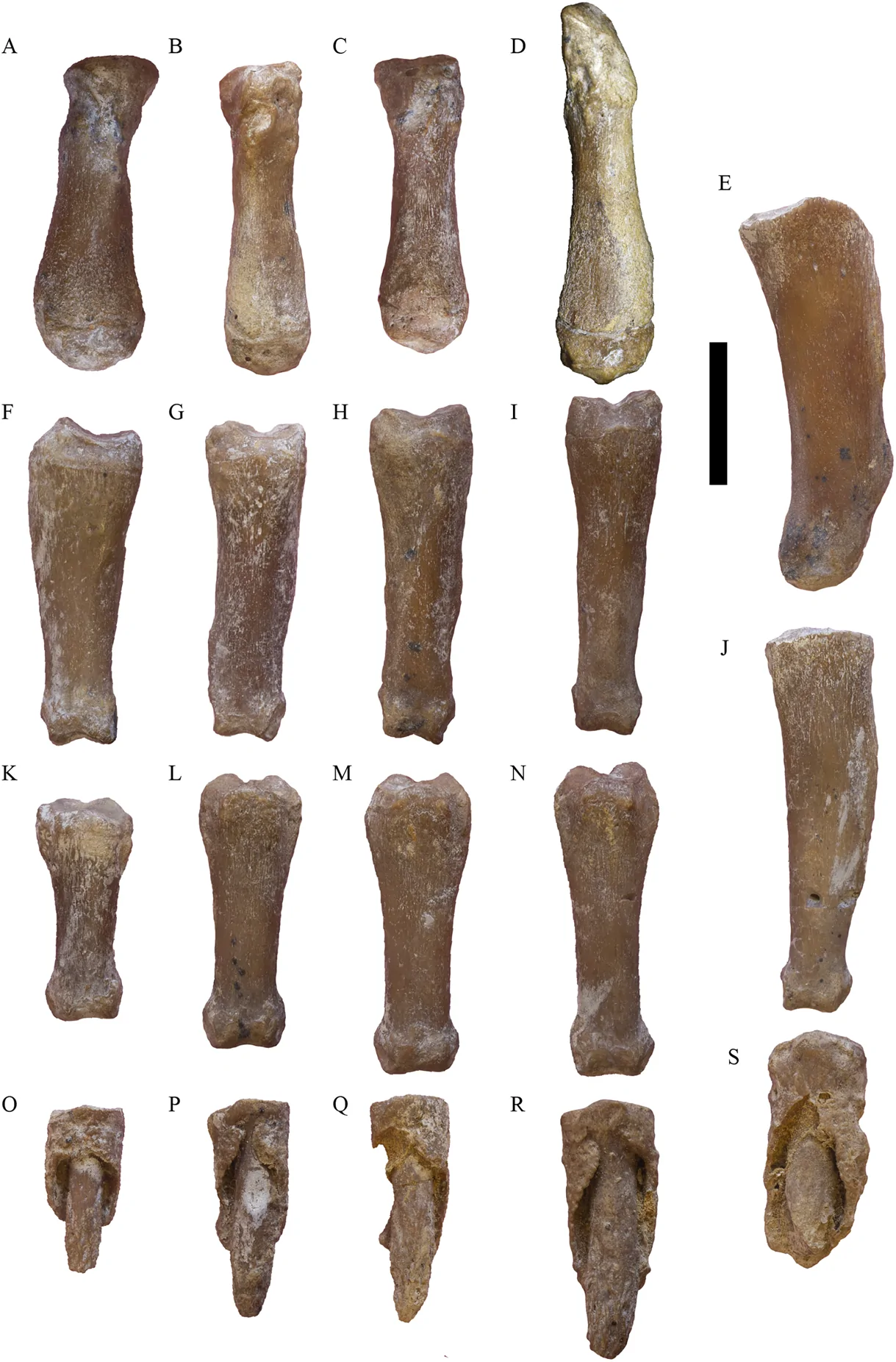
*Kawas benegasorum* (MPEF-PV 601) right metacarpal and phalanges in dorsal view. McV to McI (A–E). Proximal phalanges V to I (F–J). Intermediate phalanges V to II (K–N). Ungual phalanges V to I (O–S). Scale bar = 2 cm.

**Figure 7.**
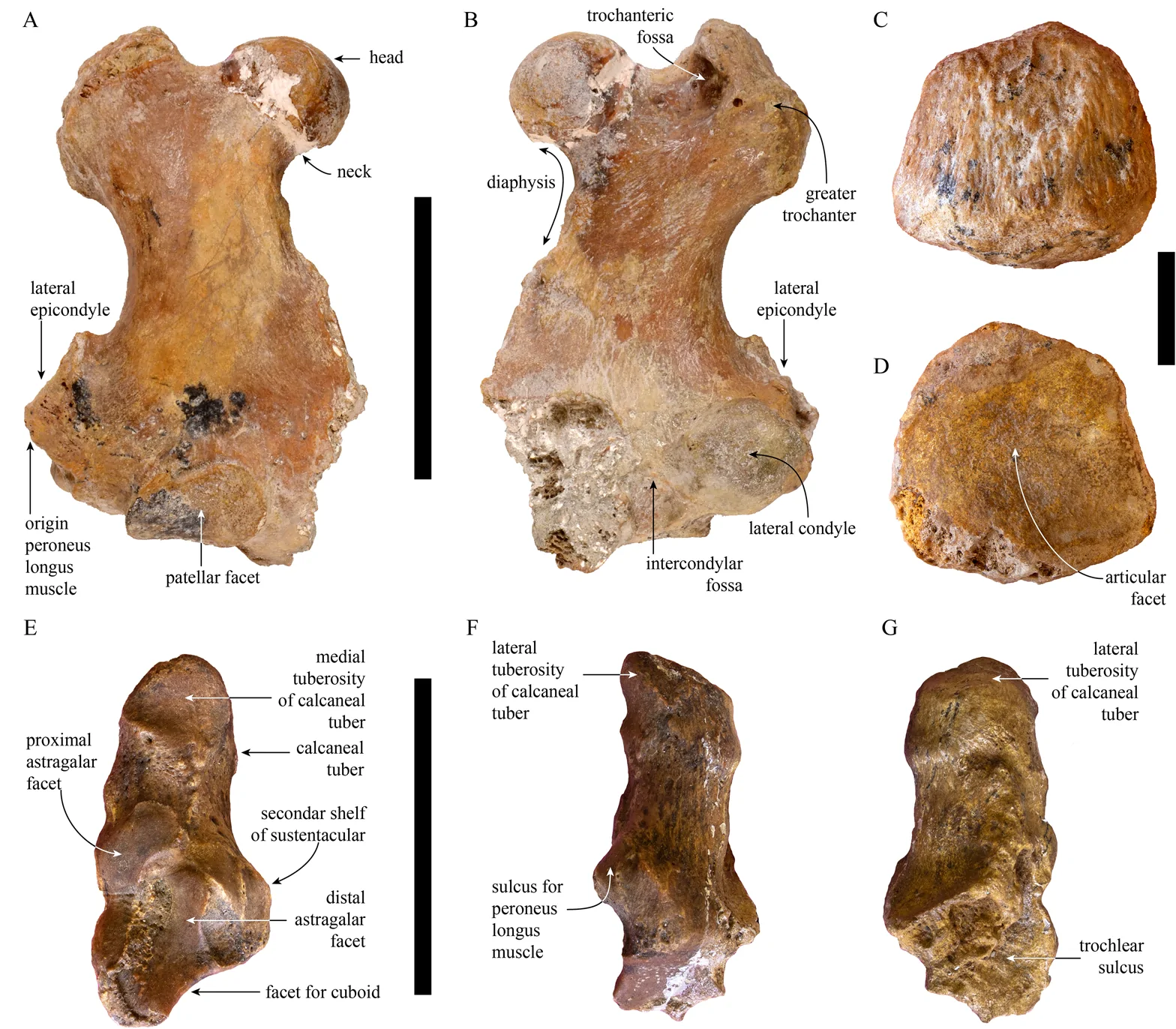
*Kawas benegasorum* (MPEF-PV 601) right hindlimb bones. Femur in anterior (A) and posterior (B) views. Patella in anterior (C) and posterior (D) views. Calcaneum in medial (E), lateral (F) and posterior (G) views. Scale bar = 5 cm in (A), (B), (E–G), and = 1 cm in (C) and (D).

**Figure 8.**
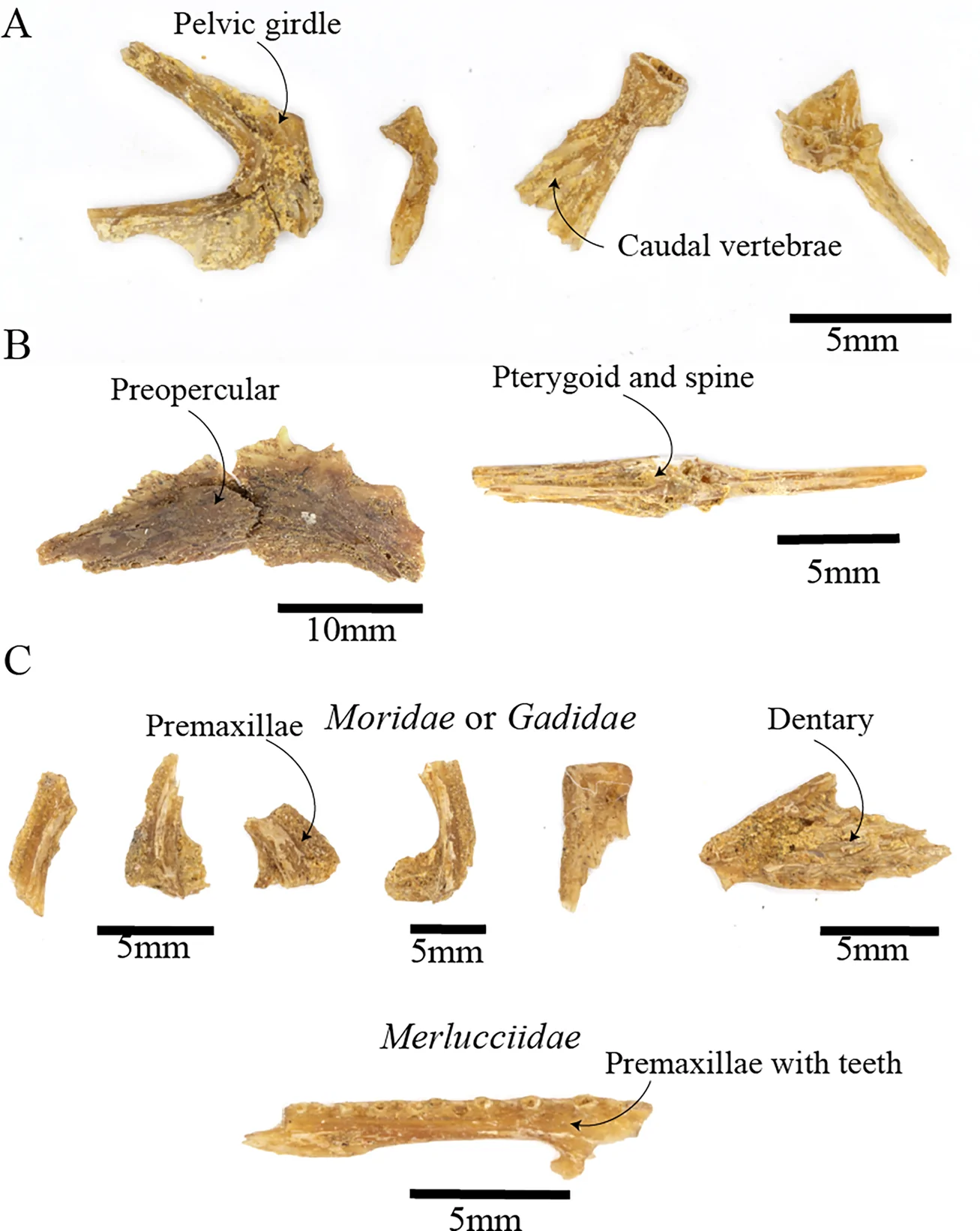
*Kawas benegasorum* (MPEF-PV 601) stomach contents. (A) Pleuronectiformes bone elements (pelvic girdle, caudal vertebra and urostyle); (B) Perciformes or Scorpaeniformes bone elements (preopercular and pterygoid); (C) Anacanthini, including representatives of Moridae, Gadidae and Merluccidae (dentary and premaxillae).

### 3.1. Holotype

MPEF-PV 601 consists of an articulate and incomplete skeleton, including three double-rooted and multi-cuspidate upper left post-canines, 12 thoracic vertebrae, five lumbar vertebrae, the sacrum, three caudal vertebrae, ribs, five complete sternebrae, the partial left and right scapula, an incomplete right humerus, the complete right radius and ulna, the distal section of the left ulna, the nearly complete right and incomplete left manus, the partial right and left innominate, the right femur and patella, the right calcaneum and entocuneiform and four posterior metatarsal and phalanges.

### 3.2. Type locality

The skeleton of *K. benegasorum* comes from the Punta San Román locality in Valdés Peninsula, Chubut province, Patagonia, Argentina.

### 3.3. Type stratigraphic horizon and age

The original level consists of bioturbated muddy sandstones accumulated in an internal shelf environment, whose specific age is estimated to be around 10 to 9 Ma (Tortonian) [21].

### 3.4. Amended diagnosis

*Kawas benegasorum* is a medium-sized pinniped (approx. 1.9–2.2 m (estimated from appendicular bone proportions reported in [31] and [32], respectively)) based on the presence of a short and robust humerus with a prominent deltopectoral crest, a short, wide and dorso-ventrally flattened femur [6]. *Kawas* is unambiguously identified as a member of Phocidae based on a femur lacking a lesser trochanter [8] and the presence of an entepicondylar foramen in the humerus [33]. It differs from members of Phocinae (except for *Nanophoca*) by having a sacrum composed of three sacral vertebrae instead of four [8]. Additionally, *Kawas* is distinguished from all other phocids owing to a unique combination of features: post-canine teeth with conical main cusps and partially fused accessory cusps (a condition seen in most extinct monachines), labial cingulum elevated below the main cusp, and the presence of a lingual shelf on the premolars; a humerus with an entepicondylar foramen (found in most phocines and specific extinct monachines like *Homiphoca* and *Magophoca*), a well-developed supinator crest (shared with phocines, such as *Monachopsis pontica*, and several monachines, including *Leptonychotes* and *Piscophoca*), and a distal insertion of the pectoralis muscle on the deltopectoral crest (typical of monachines). The radius has the bicipital tuberosity on the medial surface of the diaphysis (similar to *Pliophoca*), while the ulna features a relatively flat distal end of the styloid process (also seen in both extant and extinct monachine seals). The ungual phalanges of the fore flipper are enlarged and have variable degrees of dorsal sheath wall opening at the claw insertion site, within the range of variation observed among monachines and phocines. The gluteal fossa is moderately excavated, between the deep depression seen in some phocine seals like *Halichoerus*, *Phoca* and *Pusa*, and the faint excavation in both extant and extinct Monachinae. *Kawas* exhibits a moderate iliac eversion similar to monachine seals, and a prominent and straight femoral medial epicondylar crest that resembles the condition of the extinct *Magophoca*. Finally, the preserved pedal ungual phalange also displays intermediate characteristics between the well-developed condition in phocines and the more reduced form in monachines.

## 4. Anatomical descriptions and comparisons

The remains of *K. benegasorum* are inferred to belong to a physically mature individual (corresponding to phase III-IV in [34]) based on the fusion of the calcaneus, radius and ulna.

### 4.1. Dentition

MPEF-PV 601 preserves three double-rooted and multi-cuspidate post-canines. They correspond to a complete left M1, P4 and an incomplete P3 or P2. The roots are bulbous and elongate antero-posteriorly, with their maximum length approximately equalling that of the crown. The P3 or P2 preserves only the posterior root, which is straight and conical. In the P4, the anterior root has a straight profile, unlike the posterior, which is posteriorly arched, forming an acute angle. The anterior and posterior roots of M1 are robust, bulbous and nearly vertical.

The post-canines are characterized by crowns being mesiodistally longer than tall (table 1), especially P4 and M1. They have a relatively rugose enamel that bears faint apico-basal ridges on their buccal and lingual surfaces. This condition resembles that in *Icaphoca*, *Hadrokirus*, *Homiphoca*, *Magophoca*, *Neomonachus* and Neogene Monachinae from Argentina (MACN 3538a; [12]), Australia (NMV P254064; [35]), Chile (SGO.PV 1049a-b; [23]) and California (LACM 52624; [36]). Conversely, this condition differs from the relatively smooth enamel of *Acrophoca* and some unidentified Monachinae from the late Miocene of northern Chile (e.g. SGO.PV 21602, 22108 and 21601; [23]) and California (LACM 122584; [36]).

**Table 1.** Measurements in mm of the dentition of *Kawas benegasorum* (MPEF-PV 601).

	P2 or P3	P4	M1
height of the dorso-ventral height of the crown	6	6	6
length of the antero-posterior length of the crown	10	12	11
transverse width of the crown	6	6	6
aspect ratio of the crown	0.6	0.5	0.5

The post-canine crowns have a broad main cusp with variably developed anterior and posterior accessory cusps that are not completely separated from it. This condition is shared with several extinct monachine seals and contrasts with that of most extant and extinct Phocinae, in which the main and accessory cusps are detached. The labial surface of the crown of P2 or P3 is not fully preserved, whereas the lingual surface remains more complete. The preserved portion of the crown bears an anterior accessory cusp that is slightly taller than the remaining portion of the main cusp, whose apex is not preserved. The crown of P4 is nearly complete, although it is moderately worn on its dorsal and anterior surfaces, likely owing to the occlusion with the upper dentition. The tooth has a posterior accessory cusp that is lower than the apex of the main cusp, which constitutes the tallest point of the crown, despite the wear. In M1, the anterior margin of the main cusp lacks a discrete anterior accessory cusp, forming a continuous mesial slope that extends to the anterior edge of the crown. The apex of the main cusp of M1 is pointed posteriorly, and it is flanked distally by a well-developed posterior accessory cusp. These cusps are separated by a conspicuous intercuspidal notch. The buccal and lingual cingula are visible in all preserved post-canines, although the buccal cingulum is slightly fainter than its lingual counterpart. This arrangement differs from *Hadrokirus* and *Piscophoca*, which have equally developed buccal and lingual cingula. Instead, it resembles the condition of *Neomonachus*, MACN 3538a and NMV P254064, in which the post-canines have a prominent lingual cingulum that forms a broad but dorsally flattened shelf. In *Kawas*, the lingual cingulum also forms a subhorizontal shelf, similar to *Icaphoca*. In P2, P3 and P4, the buccal and lingual cingula have an elevation at their midpoint, below the apex of the main cusp. In M1, the buccal and lingual cingula form broad and nearly dorso-ventrally flat surfaces, with the posterior half-section forming a shallow concave basin.

### 4.2. Thoracic vertebrae

MPEF-PV 601 preserves twelve thoracic vertebrae with variable state of preservation (figure 3A,B; table 2). The vertebral bodies are barrel-shaped with subcircular anterior and posterior articular surfaces. A ventral ridge extends along the ventral margin of the vertebral body in all specimens, with variable prominence among elements. Seven pre-anticlinal and three post-anticlinal thoracic vertebrae were identified, whereas two additional vertebrae could not be assigned. Owing to the preservation state of the vertebrae, their specific position could not be determined. Therefore, we will refer to these elements as pre- and post-anticlinal vertebrae. In the anterior pre-anticlinal vertebrae, the costal fovea on the vertebral body is concave and anteriorly located on the lateral surface of the body. The prezygapophyses, postzygapophyses and neural processes are not preserved in these elements. In more posterior pre-anticlinal vertebrae, the costal fovea is small, suboval and concave, located at the anterodorsal and posterodorsal portions of the lateral surface of the body. The neural canal is triangular in the anterior view, and the neural arch is large and antero-posteriorly wide. The position and shape of the prezygapophysis show a gradual change along the anti-clinal series. In presumably more anterior elements, they are positioned at approximately the same horizontal level as the transverse processes but extend anteriorly, lying anterolateral to the anterior articular surface of the body. Their articular facets face dorsomedially. In more posterior positions, the prezygapophyses become less anteriorly extended, with their articular facet oriented more dorsolaterally. The postzygapophyses are reduced in these vertebrae and are ventrally oriented, extending posteriorly beyond the posterior articular surface. The spinous processes are elongated, tilted posteriorly and taper at the dorsal end. The costal fovea is excavated, oval, and it is located anteroventral to the transverse process in the post-anticlinal vertebrae. The prezygapophyses are well developed and positioned dorsally to the vertebral body. Their articular facets are oval and medially oriented. The transverse processes form blunt tuberosities that project lateroposteriorly. The postzygapophyses are more developed than those of the pre-anticlinal vertebrae. They are laterally inclined, with the articular surface facing laterally. The spinous processes are incompletely preserved, but the remaining section indicates they are less prominent and slightly tilted anteriorly.

**Table 2.** Measurements in mm of the thoracic vertebrae of *K. benegasorum* (MPEF-PV 601). Abbreviations: ‘?’, not preserved; ‘+’, incomplete measurement.

	pre-anticlinal							post-anticlinal			unknown position	
length	48	44	46	36	40	41	42	59	56	51	52	57
height	24	24+	23	20	26	24	31	24+	28	26	25	32
length of anterior epiphysis	43	?	?	34	30	51	?	47	?	39	45	44
height of anterior epiphysis	28	?	?	26	22	28	?	37	?	27	29	33
length of posterior epiphysis	?	?	33	22	49	?	34	?	46	38	38	47
height of posterior epiphysis	?	?	26	?	25	?	28	?	29	29	28	32

### 4.3. Lumbar vertebrae

MPEF-PV 601 preserves five lumbar vertebrae (figure 3C–F; table 3), comprising two articulated sequences even after preparation. The vertebral bodies have a sand-glass shape in ventral view and are subcylindrical in cross section. The anterior and posterior articular surfaces are oval. The prezygapophysis is well-defined, dorsolaterally arched and extends anteriorly to the anterior surface of the vertebral body. The prezygapophyseal articular facet is concave and lies anteriorly on the medial surface of the prezygapophysis. The postzygapophysis projects posteriorly and is slender and flat, with the articular facet facing ventro-laterally. Unlike the first two lumbar vertebrae (L1 and L2), where only the base of the transverse processes is preserved, the transverse processes of lumbar vertebrae L3 to L5 are nearly complete. These processes are dorso-ventrally flattened and directed anterolaterally. The spinous processes of L3 to L5 are also preserved and have a squared shape when viewed from the side. They are broad from the proximal to distal ends, and their apex is nearly straight, creating a plateau similar to that of *Acrophoca* and *Magophoca*.

**Table 3.** Measurements in mm of the lumbar vertebrae of *K. benegasorum* (MPEF-PV 601). Abbreviation: ‘?’, not preserved.

	L1	L2	L3	L4	L5
length	61	59	52	59	61
height	28	27	42	33	33
length of anterior epiphysis	40	?	40	?	?
height of anterior epiphysis	33	?	34	?	?
length of posterior epiphysis	?	45	?	?	41
height of posterior epiphysis	?	36	?	?	32

### 4.4. Sacrum

MPEF-PV 601 preserves a nearly complete sacrum that remains in articulation with the left innominate (figure 4A,B). The sacrum consists of three fused vertebrae, as in Monachinae, unlike phocine seals, which have four, except for the extinct *Nanophoca vitulinoides* [8,37]. The sacral wings are well preserved, although the left one is slightly worn in the area in contact with the iliac crest. The anterior portion of the sacrum, at the level of the sacral wings, is broad and represents the widest cross-sectional width of the sacrum, giving it a triangular shape in dorsal view (table 4). The sacral wings are ventrally curved and are laterally flared, with the ventro-lateral end extending ventrally near the level of the ventral margin of the promontory. The articular surface with the ilium is rugose and dorso-ventrally wide. It extends from the anterior portion of S1 to the half of S2 and forms a concave fossa in lateral view. The subcircular promontory in the anterior view is lateromedially wider than dorso-ventrally high. The sacral canal is broad in its anterior portion, decreasing posteriorly. The dorsomost section of the spinous process of S1 is not preserved. The spinous processes are not fused. The posterior margin of the spinous processes of S2 and S3 is anterodorsally prominent and square, contrasting with the fourth vertebra, which is trapezoidal and posteroventrally projected. The articular surface of the prezygapophyses of S1 is dorsally projected. The pre- and postzygapophyses of the sacral vertebrae are fused, forming a relatively narrow lateral sacral plate on each side. The spinous processes are not fused, similar to *Acrophoca* and differing from *Nanophoca*. Two preserved sacral foramina are on each side of the lateral sacral plate.

**Table 4.** Measurements in mm of the sacrum of *K. benegasorum* (MPEF-PV 601).

total width across the anterior portion	73
height of the anterior articular facet (promontorium)	34
width of the anterior articular facet (promontorium)	44
width across the wings	118
width between the prezygapophyses	47

### 4.5. Caudal vertebrae

The first, second and third caudal vertebrae of *Kawas* are preserved. They progressively decrease in size (figure 3G,H; table 5). The neural spine of the first and second vertebra is dorso-ventrally low and thick in dorsal view. The transverse processes of the first vertebra are robust and elongated antero-posteriorly, giving the vertebra a square appearance in the dorsal view. The prezygapophyses are well-defined, and the postzygapophyses project posteriorly.

**Table 5.** Measurements in mm of the caudal vertebrae of *K. benegasorum* (MPEF-PV 601). Abbreviation: ‘-’, not applicable.

	C1	C2	C3
length	34	37	34
height	24	17	24
length of anterior epiphysis	24	18	-
height of anterior epiphysis	22	21	-
length of posterior epiphysis	18	20	-
height of posterior epiphysis	20	21	-

### 4.6. Sternum

Five posterior sternebrae are preserved. These elements are short and broad with rugose articular surfaces. The sternebrae are antero-posteriorly short and hourglass-shaped with a spongy surface. Their articular surfaces are rugose and suboval. The last sternebra is more antero-posteriorly elongated than the anterior ones. It is flattened and also hourglass-shaped. It bears tubercles with posterolateral articular surfaces for the ribs in the anterior part. The middle section is subcylindrical and widens transversely posteriorly to form a rounded posterior articular surface.

### 4.7. Ribs

MPEF-PV 601 preserves a complete set of 12 left and eight right ribs. Most of the left ribs are complete, with the first and third ribs missing the proximal region and the fourth rib missing the head. The first three or four right ribs are not preserved; the next four are missing the proximal region. The ribs become increasingly curved in anterior and posterior views and generally bear a small tubercle relative to an enlarged and rounded head, forming a faint protuberance at the base of the neck when preserved. The head becomes increasingly more prominent and spherical in the posterior ribs. The insertion area for the external intercostalis is well-marked on the dorsal surface.

### 4.8. Scapula

MPEF-PV 601 preserves the incomplete left and right scapulae (table 6). The incomplete glenoid cavity and the caudal half of the left scapula, and the caudal portion of the right scapula are preserved. The glenoid is suboval and moderately concave, similar to *Magophoca*. Although incompletely preserved, the neck is relatively well developed but short, comparable to *Homiphoca* and *Piscophoca*. The base of the scapular spine and acromion are incompletely preserved and are located approximately at the midpoint of the glenoid cavity. Still, their base suggests they were relatively low. The inferred position of the scapular spine suggests that the surface area of the supraspinatus region makes up nearly half of the scapula's surface in posterior view, resembling the condition seen in Monachinae, and differing from Phocinae, where the surface area for the supraspinatus is smaller and closer to that of the infraspinatus and teres major muscles [8]. The infraspinatus fossa is slightly concave in lateral view and is bounded by a low ridge marking the insertion of the teres major muscle.

**Table 6.** Measurements in mm of the scapula of *K. benegasorum* (MPEF-PV 601). Abbreviation: ‘+’, incomplete measurement.

preserved total length	153
preserved total width	134
preserved articular surface width	32+
preserved total articular surface length	28
length of the glenoid region	31+
width of the glenoid region	27

### 4.9. Humerus

MPEF-PV 601 consists of the distal two-thirds of the right humerus (figure 5E–H; table 7). Although the proximal portion is not preserved, the remaining section suggests that the humerus is proximodistally short and robust, as seen in phocids. In anterior and posterior views, the humerus has a slender appearance compared with some extinct (e.g. *Acrophoca*, *Homiphoca*, *Piscophoca* and *Pliophoca*) and extant (e.g. *Hydrurga*, *Leptonychotes* and *Neomonachus*) Monachinae, but a slightly more robust aspect than *Australophoca*, *Magophoca*, an undetermined Neogene phocid from Argentina (e.g. MER 685, previously identified as *Properiptychus argentinus* by [12]) and extinct Phocinae, such as *Leptophoca* and *Nanophoca*. The deltopectoral crest is elongated in lateral view, reaching the distal third of the shaft, similar to Monachinae (except for *Leptonychotes* and *Lobodon*) and unlike most Phocinae in which this crest is limited to the proximal half of the humerus. It is gently curved and gradually tapers distally to the diaphysis, as in Monachinae, and differs from extant phocine seals in which the deltopectoral crest terminates abruptly. Although MPEF-PV 601 lacks the proximal first third of the humerus, the preserved section of the deltopectoral crest suggests that this structure had a more curved outline in lateral view, a feature characteristic of Monachinae seals and in contrast to the straighter and more rectangular profile of extant and extinct Phocinae [38]. The insertion area for the pectoralis muscle is rugose and is located in the distal section of the deltopectoral crest, differing from living and extinct Phocinae (see [39]). Compared with *Acrophoca*, *Pliophoca*, to some extent *Piscophoca*, and *Neomonachus*, the supinator crest is more prominent, giving the humerus a contoured appearance in lateral view, similar to the extinct *Australophoca*, *Homiphoca* and *Magophoca*, and the extant *Lobodon* and, to a lower degree, some unidentified Monachinae (e.g. USNM 181419; [38]). However, the supinator crest is notably less developed than in extant and extinct Phocinae. The medial epicondyle is relatively straight, resembling the configuration of *Magophoca*, but differing from the extinct *Acrophoca* and *Australophoca* and the extant *Hydrurga*, *Leptonychotes*, *Lobodon* and *Mirounga*, in which the medial epicondyle is less developed, and from *Pliophoca*, in which it is mediodistally oriented. The lateral epicondyle is laterally expanded with its longer axis vertically straight. It gives this area a trapezoidal shape in anterior and posterior views, similar to *Acrophoca*, *Piscophoca*, *Neomonachus*, *Leptonychotes*, and extant and extinct phocine seals, such as *Monachopsis*. MPEF-PV 601 preserves a prominent and oval entepicondylar foramen as seen in extant and extinct Phocinae and the extinct Monachinae such as *Homiphoca* and *Magophoca*. The olecranon and coronoid fossa are relatively shallow, although a slightly deeper concavity marks the latter. The trochlea in the distal epiphysis is well-preserved and crescent-shaped in the posterior view.

**Table 7.** Measurements in mm of the humerus of *K. benegasorum* (MPEF-PV 601). Abbreviation: ‘+’, incomplete measurement.

proximal-distal length	101+
distal length of the epicondyle of the humerus (medial-lateral length)	46
maximum preserved width of the deltopectoral crest of the humerus (anterior-posterior)	32
maximum preserved length of the deltopectoral crest of the humerus (proximal-distal)	51
distal width trochlea	26
anterior width of the trochlea	28
transverse width mid-diaphysis	24
antero-posterior thickness of the medial condyle	22
antero-posterior thickness of the lateral condyle	25
diameter mid-diaphysis and deltopectoral crest in lateral view	44

### 4.10. Radius

The right radius is fully preserved (figure 5I,J). The radius is shortened proximodistally (table 8), transversely flattened and flaring at the distal end, characteristic of phocids. The proximal epiphysis is subhorizontal and slightly convex in lateral and medial views. The lateral portion of the proximal epiphysis is laterodistally sloped, similar to *Acrophoca*, but differing from *Magophoca* and *Pliophoca*, in which this region is flat and nearly horizontal. The articular facet of the humerus is slightly concave and rounded in the proximal view, similar to *Acrophoca*. The medial margin of the articular facet for the humerus contacts the facet for the ulna, differing from *Homiphoca*, *Magophoca* and *Pliophoca*, in which the articular facet for the ulna forms a continuous posterolaterally oriented distal lip. The bicipital tuberosity is located medially on the medial surface of the diaphysis and is well-defined and separated from the proximal epiphysis. It is oval, and its surface is ridged. It is located more proximally than in *Acrophoca* and *Piscophoca*, resembling *Pliophoca*, referred material to *Australophoca* (MUSM 569/SAS 989), and different from extant Monachinae, where the bicipital tuberosity is posteromedially. It also varies from phocine seals, in which the bicipital tuberosity is more medial and rounded. The insertion area for the radio-ulna ligament in the posterior margin of the diaphysis is poorly developed, as in *Acrophoca*, differing from the conspicuous elongated tuberosity present in *Pliophoca*, represented by a shallow depression. The anterior margin of the diaphysis is smooth and lacks prominent ridges for muscle insertion. The insertion area for the pronator teres muscle forms a modest prominence in the mid-section of the diaphysis. The insertion area for the brachioradialis muscle forms a longitudinal and nearly vertical ridge in the distal third of the diaphysis. It differs from the distinctly convex tuberosity present in *Magophoca*, resembling the configuration of *Acrophoca* and *Piscophoca*. Grooves for the tendon are visible in the distal portion of the lateral surface of the radius. The groove for the extensor digitorum lateralis muscle tendon is broader, deeper and relatively better delineated than that for the extensor digitorum communis muscle tendon, opposing the configuration of *Acrophoca*, *Magophoca*, *Piscophoca* and MUSM 569/SAS 989. The groove for the tendon of the abductor pollicis longus muscle on the anterior margin of the diaphysis is deep and laterally covered by a tiny bony lip. The articular facet for the scapho-lunar is concave, similar to *Magophoca* and *Pliophoca*, but smaller than the latter. The articular facet of the ulna in the mediodistal section of the radius forms a conspicuous rounded projection.

**Table 8.** Measurements in mm of the radius of *K. benegasorum* (MPEF-PV 601).

total length	136
height of the proximal epiphysis	16
width of the proximal epiphysis	22
height of the distal articular facet	18
width of the distal articular facet	25
minimum diameter of the neck	16
length of the scapho-lunar facet	22
antero-posterior length of the distal articular facet	52

### 4.11. Ulna

The complete right ulna and the distal part of the left ulna, including the styloid process, are preserved (figure 5K–M; table 9). In lateral and medial views, the ulna is relatively short, broad and slightly curved posteriorly, especially in its distal half. It resembles *Acrophoca*, *Piscophoca* and referred material to *Australophoca* (MUSM 569/SAS 989). It differs from the straighter profile of the extinct Monachinae *Magophoca*, *Homiphoca* and *Pliophoca*, as well as the extinct *Nanophoca* and *Leptophoca*. The olecranon process is well developed and transversely flat, and its cranial margin is straight, as described in *Acrophoca*, but differs from the marked concavity of this region in *Australophoca* and *Magophoca*. The lateral margin of the olecranon process is triangular and forms an acute angle in lateral and medial views, similar to *Piscophoca*. It differs from the more triangular shape of the olecranon process observed in extant phocine (e.g. *Phoca*) and the more rounded configuration in some extant Monachinae (e.g. *Hydrurga* and *Leptonychotes*). The anconeal process (= anconeal tuberosity in [40]) is well defined. It forms a medially oriented triangular projection in the anterior view, similar to extinct monachine seals and members of the Phocinae. The proximal half of the shaft is straight, although the distal portion is slightly curved laterally in the anterior view. The greater sigmoid cavity for articulation with the humerus is slightly taller than transversally broad, and its anterior surface is concave. This cavity's cranial and lateral margins are rounded, unlike *Magophoca* and *Pliophoca*, where the cranial margin is saddle-shaped. The lesser sigmoid cavity for articulation with the radius is flatter than the adjacent cavity, has an anteromedially oriented semilunar shape, and is longitudinally longer than transversally wide in the anterior view. The medial surface of the shaft is shallowly excavated, forming a relatively continuous but progressively narrowing depression from the olecranon to the caudal section of the ulna to insert the flexor muscles. The insertion site for the abductor pollicis longus on the lateral surface of the ulna is shallowly excavated. The oblong rugosity is a distinct, medially oriented ridge located in the middle portion of the bone in the anterior view, between the distal margin of the lesser sigmoid cavity and the styloid process. The styloid process is distinctive and relatively flat, similar to extant and extinct members of the Monachinae, but it differs from some phocine seals, where the shape of this structure ranges from pointed (e.g. *Monachopsis* and *Nanophoca*) to flattened (e.g. *Leptophoca*). The ulnar notch is preserved, creating a gentle concavity when viewed laterally.

**Table 9.** Measurements in mm of the ulna of *K. benegasorum* (MPEF-PV 601). Abbreviation: ‘?’, not preserved.

	Right	Left
total length	146	?
greater sigmoid facet length	17	?
greater sigmoid facet width	18	?
lesser sigmoid facet length	15	?
lesser sigmoid facet width	13	?
sigmoid facet length	37	?
sigmoid facet width	22	?
oblong rugosity width	9	9
height of the olecranon crest above the trochlear or sigmoid notch	54	?

### 4.12. Manus

MPEF-PV 601 preserves the nearly complete right anterior autopodium, including the carpals (i.e. scapholunar, trapezoid, trapezium, magnum and two unidentified bones), metacarpals and phalanges, and the incomplete left manus, consisting of the pisiform, metacarpal I, phalange I, proximal phalange II and an intermediate phalange (figure 6; table 10).

**Table 10.** Measurements in mm of the elements of the manus of *K. benegasorum* (MPEF-PV 601). Abbreviations: ‘+’, incomplete measurement; ‘?’, not preserved.

right metacarpals	Mc I	Mc II	Mc III	Mc IV	Mc V
total length	63+	56	45	44	46
maximum width proximal end	21	15	14	12	14
dorso-ventral diameter proximal end	16	11	11	11	10
maximum width distal end	14	12	11	14	14
dorso-ventral diameter distal end	9	6	7	10	11
minimum transversal midshaft diameter	10	7	7	9	9
**right proximal phalange**	**Ph. I**	**Ph. II**	**Ph. III**	**Ph. IV**	**Ph. V**
total length	61+	50	47	45	41
maximum width proximal end	18	14	14	16	15
dorso-ventral diameter proximal end	12	10	11	12	12
maximum width distal end	12	11	11	12	13
dorso-ventral diameter distal end	7	6	7	7	11
minimum transversal midshaft diameter	8	6	7	7	10
**right intermediate phalange**	**Ph. I**	**Ph. II**	**Ph. III**	**Ph. IV**	**Ph. V**
total length	?	42	39	35	29
maximum width proximal end	?	14	14	14	13
dorso-ventral diameter proximal end	?	10	10	10	10
maximum width distal end	?	12	12	11	10
dorso-ventral diameter distal end	?	5	7	6	6
minimum transversal midshaft diameter	?	6	6	6	5
**right ungual phalange**	**Ph. I**	**Ph. II**	**Ph. III**	**Ph. IV**	**Ph. V**
total length	29	30	31	27	21
maximum width	13	12	12	11	11
**left anterior autopodium**	**Mc I**	**Ph. I**	**Ph. 2**	**Ph. ?**	
total length	73	61	56	35	
maximum width proximal end	20	18	12	14	
dorso-ventral diameter proximal end	17	12	16	10	
maximum width distal end	14	12	15	11	
dorso-ventral diameter distal end	9	8	9	6	
minimum transversal mid-shaft diameter	11	7	9	6	

The radial facet of the scapholunate bone is concavo-convex with no constriction between its two portions. A ridge separates the unciform facet from the subcircular magnum facet; the trapezoid facet is squoval, while the trapezium facet is oval. The trapezoid bone is subtriangular in proximal view with the scapho-lunar facet slightly convex. The trapezium facet is flat, oval and smaller than the adjacent flat and rectangular facet for the metacarpal II. The trapezium bone bears a concave, dorsally elevated trapezoid facet. The scapho-lunar facet is subvertical and convex in dorsal and palmar views. These facets converge at an elevated ridge, with its apex anteriorly located. The dorsal surface has a conspicuous antero-posterior sulcus. The metacarpal I facet is suboval and concavo-convex, whereas the metacarpal II facet is rounded and convex, forming an almost right angle with the trapezoid facet. In the mediodistal view, the metacarpal I facet is intermediate between the heart-shaped phocines and the trifoliate morphology of *Magophoca* [8]. The magnum bone is squared in medial view, with the metacarpal II and metacarpal III facets forming flat and rectangular surfaces separated by a ridge. The unciform facet is oval, and the scapho-lunar facet is convex.

The metacarpals are keeled in their distal end, resembling Phocinae and some extinct Monachinae, such as *Magophoca*. The metacarpal I lacks its proximal epiphysis; however, the preserved section of this metapodial has roughly the same proximodistal length as the metacarpal II. Still, if the proximal epiphysis were preserved, it is likely that the metacarpal I would have been longer than the metacarpal II, similar to extant and extinct Monachinae and contrasting with the approximately equivalent length of metacarpals I and II of phocine seals [8]. In both dorsal and palmar views, the distal part of this metapodial is slightly bent medially, which partially resembles *Magophoca* and *Piscophoca* and partially *Acrophoca*. In the latter, metacarpal I varies from a generally straight form to a more bent appearance. Unlike the more squared aspect of *Acrophoca* and *Piscophoca*, the distal section of the metacarpal I of MPEF-PV 601 is rounded. The distal ends of metacarpals II-V are rounded, resembling those of *Acrophoca*, *Magophoca* and *Piscophoca*. The facet for the trapezoid is distinctly marked and lateromedially deflected in the dorsal view in metacarpal II, similar to *Acrophoca* and *Pliophoca*. The facets for metacarpal II and metacarpal IV are nearly flat in dorsal view in metacarpal III, similar to *Piscophoca* and *Pliophoca*, and differing from the laterally expanded facet for metacarpal II in *Magophoca*. The facet for the unciform in the metacarpal IV is flat and nearly horizontal. It has a squared aspect, differing from the more rounded end of *Pliophoca* and the subhorizontal and trapezoidal shape of *Magophoca*. The facet for the metacarpal IV, in the proximal section of the metacarpal V, is less developed than in *Acrophoca* and does not form a medial projection.

The metacarpophalangeal and interphalangeal articulations are trochlear and plantar-distally flatter, broader and hinge-like, as in monachines, relative to the more rounded trochlea observed in extant and extinct phocine seals. The anterior surface of the proximal phalanges II-V is concave for articulating the palmar keel of the distal end of the corresponding metacarpals. The proximal end of phalange I is nearly flat, contrasting with the more rounded shape seen in *Piscophoca*. The diaphysis of the intermediate phalanges is flattened and oval in cross section, differing from the somewhat rounded configuration in phocine seals. The intermediate phalanges II through IV are almost the same length, while the intermediate phalanx V is shorter. This arrangement resembles the morphology found in Phocinae and some extinct Monachinae, such as *Acrophoca*, *Australophoca* and *Pliophoca*. However, it differs from the significant shortening observed in extant Monachinae [33]. The specimen MPEF-PV 601 retains the entire sequence of ungual phalanges, which are dorso-ventrally flattened, have short epiphyses and form enlarged bony claws. These claws are larger than those of most extant and extinct monachine seals, except for *Acrophoca* and *Magophoca*, in which claws are enlarged and approach the claw morphology of phocine seals. The ungual phalanges also show a variable degree of dorsal opening of the sheath wall at the claw insertion, consistent with the dorsally open condition in most monachines and the closed but variable state reported in phocines [8].

### 4.13. Innominate

The incomplete right and left innominates are preserved, with the left cemented with the sacrum (figure 4C–F). For both innominates, most of the iliac section of the pubis and the distal half of the ischium are missing. The ilium is antero-posteriorly short (table 11), measuring approximately twice the acetabular diameter. This proportion is similar to that observed in certain extant (e.g. *Hydrurga*), extinct monachines (e.g. *Acrophoca* and *Magophoca*) and extant phocine seals (e.g. *Phoca*). However, it differs from the more antero-posteriorly elongated ilium of *Piscophoca*. The ilium is anteriorly flared and resembles the general appearance of the extinct *Acrophoca*, *Piscophoca* and MUSM 569/SAS 989, the extant *Hydrurga*, *Lobodon*, *Neomonachus* and *Ommatophoca* and phocine seals like *Halichoerus* and *Phoca*. The iliac crest and the anterodorsal and anteroventral processes are well-developed, similar to MUSM 569/SAS 989. Unlike Phocinae, in which the anterodorsal process forms an acute angle, this process forms an obtuse but nearly right angle in *Kawas* that mirrors the condition of *Acrophoca*, *Piscophoca*, *Hydrurga* and *Neomonachus*. The anteroventral process is preserved in the right innominate; it is well-developed, and it forms an acute angle, similar to *Acrophoca*, *Hydrurga* and phocine seals. *Kawas* also exhibit developed posteroventral and posterodorsal processes, which are shared with Phocinae and some extant and extinct Monachinae. The gluteal fossa is visible and has an intermediate state between the distinctive depression in some phocine seals (e.g. *Halichoerus*, *Phoca* and *Pusa*) and the faint excavation in MUSM 569/SAS 989, *Hydrurga* and *Neomonachus*. The ilium is laterally everted, reaching approximately 62°, corresponding to the range of extant and extinct Monachinae and differing from the more laterally tilted condition of extant and extinct Phocinae. The acetabulum is enlarged, and its dorsal margin contributes to the dorsal margin of the innominate, similar to *Acrophoca*, *Magophoca*, *Piscophoca* and phocine seals. It differs from the more centred position observed in *Homiphoca*, MUSM 569/SAS 989, *Hydrurga*, *Leptonychotes* and *Neomonachus*. The acetabulum is deeply excavated, and its dorsal margin is raised over the horizontal surface of the bone. The iliopectineal eminence forms a conspicuous anteroventrally oriented hook, ventral to the mid-level of the acetabulum, similar to *Homiphoca*. The iliopectineal eminence in *Kawas* partially resembles the condition of *Acrophoca*, which is pronounced but more anteriorly directed, and differs from the moderately pronounced condition in the extinct *Piscophoca*, MUSM 569/SAS 989 and the extant monachine *Hydrurga*, *Leptonychotes* and *Neomonachus*. The proximal section of the ischium is lateromedially flattened. The ischial spine is well developed and forms a tapering projection, similar to *Acrophoca*. Only the base of the iliac branch of the pubis is preserved. The right innominate has a distinctive, suboval and separated obturator foramen located approximately1.5 cm posterior to the ventro-lateral margin of the acetabulum. This foramen is absent in the left innominate. Although the absence of the area of the pelvic symphysis prevents us from having a more certain assessment of the shape of the post-acetabular section, the preserved section of the ischium and the base of the iliac branch of the pubis suggest that the obturator window was antero-posteriorly elongated and dorso-ventrally narrow, and presumably similar to *Piscophoca*, *Neomonachus* and phocine seals.

**Table 11.** Measurements in mm of the left and right innominate of *K. benegasorum* (MPEF-PV 601). Abbreviation: ‘+’, incomplete measurement.

	Left	Right
acetabulum diameter	27	28
total height at the level of the acetabulum	47	45
total posterior length from acetabulum	122+	137
height of the gluteal fossa	68	73
total antero-posterior length	173+	191

### 4.14. Femur

The right femur is preserved (figure 7A,B; table 12). It is proximodistally short and antero-posteriorly flat, as in Phocidae. The femoral head is rounded but less globular than *Australophoca*, *Magophoca*, *Pliophoca* and most extant and extinct Phocinae. The femoral head and neck are oriented proximomedially. The neck is relatively short and slightly flattened in cross section, giving it a relatively robust aspect to the proximal region of the femur, similar to *Acrophoca* and *Homiphoca*. The greater trochanter is predominantly rectangular, with a curved dorso-lateral edge and a pointed proximal end that mirrors the aspect of *Pliophoca*. In the anterior and posterior views, the greater trochanter is higher than the femoral head, similar to the configuration of most extinct (e.g. *Acrophoca*, *Australophoca* and *Magophoca*) and some extant (e.g. *Neomonachus* and *Ommatophoca*) monachines and extant (e.g. *Cystophora*, *Pagophilus* and *Pusa*) and extinct Phocinae (e.g. *Monachopsis*). The greater trochanter has a deep and distinctive trochanteric fossa on its posterior surface, partially covered by a bony flap at its proximal edge. The presence of a trochanteric fossa is shared with most extinct Monachinae and extant and extinct phocines. The narrowest part of the femur is slightly proximal to the midpoint of the diaphysis, contrasting with some extinct phocines, such as *Monachopsis*, in which the narrowest point is located in the first-third of its length. The epicondylar crest is well-defined, resembling the aspect of some extant (e.g. *Phoca*) and extinct (e.g. *Nanophoca*) Phocinae and Monachinae like *Hydrurga* and *Ommatophoca*. The epicondylar crest is rugose and sub-vertical. Its proximal edge reaches the middle section of the femur, just below the narrowest section (approx. 1 cm below the level of a nutrient foramen in the medial margin). The nutrient foramen is located dorsal to the proximal end of the epicondylar crest, similar to the condition observed in *Magophoca* [8]. The postero-medial region of the last third of the femur, corresponding to the position of the medial distal condyle, is not preserved, preventing inferences about the length and distal development of the epicondylar crest. Nevertheless, the development of the preserved portion of the medial epicondylar crest gives the femur a lateral contortion in its distal section, giving the femur a similar aspect to extant Monachinae, but less pronounced than in Phocinae. The lateral epicondyle is well developed, as seen in Monachinae, and differs markedly from Phocinae. It forms a proximodistally sloped rugose crest whose anterior edge is pointed and is located under (approx. 1 cm) the level of the equivalent level of the epicondylar crest. This configuration differs from some monachine seals, like *Magophoca* and *Pliophoca*, in which the proximal end of the lateral epicondyle is near the level of the epicondylar crest. The lateral epicondyle is laterally expanded, which, together with the preserved region of the medial epicondylar crest, gives a flared appearance of the distal third of the femur relative to some other extinct (e.g. *Acrophoca*, *Homiphoca*, *Magophoca* and *Pliophoca*) and extant (e.g. *Leptonychotes*, *Lobodon* and *Ommatophoca*) Monachinae and extant Phocinae (e.g. *Phoca*). The insertion site for the peroneus longus muscle is grooved and located on the anterolateral surface of the distal lateral epicondyle, similar to most monachinae seals. The medial distal condyle is not preserved. The lateral distal condyle is suboval and slightly convex. The intercondylar fossa on the posterior surface of the femur is flared distally. The suprapatellar facet is indistinguishable. The patellar facet on the anterior surface is wider lateromedially than it is high proximodistally. This configuration resembles the characteristics of both extant and extinct Monachinae, except for *Acrophoca*, which has a patellar facet nearly equal in width and height, contrasting with the Phocinae, which exhibit a patellar facet taller than it is wide.

**Table 12.** Measurements in mm of the femur of *K. benegasorum* (MPEF-PV 601).

width of the head	19
height of the head	21
maximum width of the proximal epiphysis	55
distance between the head and the trochanter	14
maximum diameter of the trochanter	24
minimum diameter of the trochanter	14
width of the lateral condyle	27
height of the lateral condyle	22
width of the patellar facet	26
height of the patellar facet	18
total proximo-distal length	99
antero-posterior width of the diaphysis	19
latero-medial width of the diaphysis	30

### 4.15. Patella

The right patella is slightly wider (24 mm) than higher (23 mm), in contrast to the higher-than-broader aspect of *Magophoca* (figure 7C,D). The most proximal part narrows to form an asymmetric peak-like edge, giving the patella a hexagonal shape. The anterior surface is rugged and convex in lateral view. Conversely, the articular facet is predominantly squared, and its surface is smooth and slightly concave. In lateral view, the patella has a conical appearance, reaching a maximum thickness of 13 mm.

### 4.16. Calcaneum

The complete right calcaneum is preserved in *K. benegasorum* (figure 7E–G). The height/length ratio of the calcaneum is approximately 0.46, which is lower than in other extinct Monachinae and extant Lobodontini ([8]; table 13). On the medial surface, facets for articulation with the astragalus and fibula are well developed and visible, in contrast to the general condition observed in most extant phocine and monachine seals, in which facets are relatively less defined. Specifically, the ectal or proximal astragalar facet is sickle-shaped and convex in the area where it articulates with the astragalus, which is continuous with the fibular facet, which is suboval and tapered. These two facets align in the medial plane, similar to *Acrophoca* and *Magophoca*. The sustentacular facet is lengthened and narrow along its longer axis, with the distal end of the astragalus slightly wider. This configuration contrasts with the shorter sustentacular facet observed in some phocine seals like *Halichoerus* and the more rectangular condition in *Phoca* and *Pusa*. The elongated sustentacular facet in *Kawas* also differs from extant monachine seals such as *Hydrurga* and *Lobodon*, and, to a lower level, from the relatively short facet in the extinct *Acrophoca*, *Piscophoca*, *Pliophoca* and extant Monachinae, such as *Neomonachus*. The sustentacular facet widens conspicuously distally, and the contact area with the cuboid facet is expanded dorsally, similar to *Magophoca* but differing from *Pliophoca*. The distal end of the distal astragalar facet is rounded and oriented medially within a well-developed secondary sustentaculum shelf. The cuboid articular facet is concave and square-shaped in proximal view.

**Table 13.** Measurements in mm of the calcaneum of *K. benegasorum* (MPEF-PV 601).

maximum length	61
maximum transverse diameter	26
maximum dorso-ventral height	28

### 4.17. Entocuneiform

MPEF-PV 601 preserves the right entocuneiform with a well-developed plantar process, as in monachines, unlike the less developed condition in Phocinae (see [8] and references therein).

### 4.18. Metatarsal and phalanges

MPEF-PV 601 preserves the right metatarsal I, intermediate phalanges I and V and ungual phalanx V. The elements are nearly entirely preserved and decrease in proximodistal length (table 14). Metatarsal I is relatively shorter than those of extant and extinct monachines, within the range of extant phocines [8]. The diaphysis is slightly laterally curved, and the facet for the articulation with the entocuneiform is concave. The diaphyses of the intermediate phalanges are somewhat dorso-ventrally flattened, contrasting with the more rounded cross section in phocine seals. The proximal and distal epiphyses are simple, with the proximal articular surface being slightly concave. The distal articular surface of both phalanges is intermediate between the saddle-shaped condition of Monachinae and the cupped articular surfaces of Phocinae. The ungual phalange is unreduced, dorso-ventrally flattened and its distal half, in the area of the bony claw, is characterized by having conspicuous rugosities.

**Table 14.** Measurements in mm of the metatarsal and posterior phalanges of *K. benegasorum* (MPEF-PV 601). Abbreviation: ‘?’, not preserved.

	Mt I	Ph. I	Ph. V	ungual Ph. V
total length	84	71	54	56
maximum width proximal end	24	19	14	20
dorso-ventral diameter proximal end	17	14	11	?
maximum width distal end	16	14	10	?
dorso-ventral diameter distal end	12	9	6	?
ninimum dorso-ventral midshaft diameter	11	8	6	?

### 4.19. Stomach contents

Several dermal and skeletal remains resembling small- to medium-sized bony fish were recovered in association with the skeleton of MPEF-PV 601, within the abdominal region [5]. They were carefully prepared, curated and stored in glass vials (figure 8). The stomach contents of *K. benegasorum* were initially described by Cozzuol [5], who recognized remains of ‘one angel shark (*Squatina* sp.) and four species of teleost fishes'. However, unlike the original report, no *Squatina* remains were identified during our study. Instead, our examination resulted in the recognition of several osteological elements consistent with the morphology of representatives of Pleuronectiformes (pelvic girdle and caudal vertebra with urostyle with affinities to Bothidae; figure 8A), Perciformes or Scorpaeniformes (pterygoids, dorsal spines and preoperculars with affinities to Serranidae or Scorpaenidae; figure 8B) and Gadiformes (premaxillaries with the insertion of the teeth, dentary with teeth with affinities to Gadidae or Moridae, premaxillaries with the insertion of the teeth with affinities to Merlucciidae; figure 8C; supplementary material, table S2). Additional bony elements are also considered part of the stomach content; however, owing to their fragmentary condition, it is not possible to make an anatomical identification.

### 4.20. Phylogenetic analysis

The equal-weight parsimony analysis recovered 7708 most parsimonious trees (MPTs) of 644 steps, resulting in a poorly resolved strict consensus tree (supplementary material, figure S1). The implied-weight analysis under K = 3 recovered nine MPTs (fit = 64.24), with a retention index (RI) of 0.612 and a consistency index (CI) of 0.307. The analysis improved the overall resolution relative to the strict consensus tree obtained under equal weight, especially among extant species of Monachinae and Phocidae. The resulting strict consensus tree (figure 9) recovered Phocidae as a monophyletic group, with *Devinophoca claytoni* as a sister taxon. Within Phocidae, the subfamilies Phocinae and Monachinae were recovered, with *Kawas* placed within Phocinae, where it formed a polytomy with *Devinophoca emryi*, ‘*Leptophoca proxima*’, and a clade comprising the remaining phocines. Overall, most nodes showed low support values, with a few exceptions.

**Figure 9.**
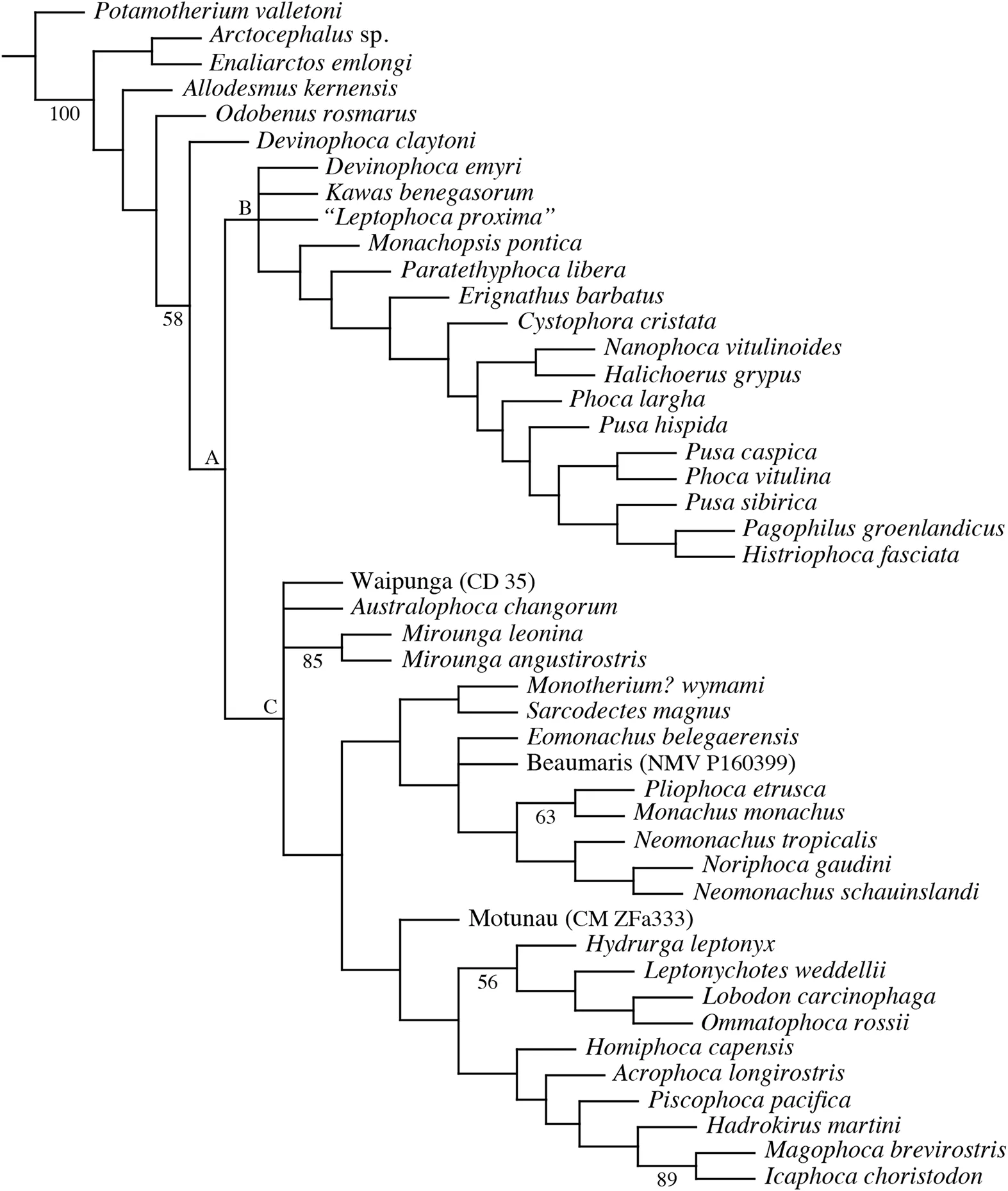
Strict consensus of nine most parsimonious trees resulting from the phylogenetic analysis with implied weighting (K = 3). Numbers below the nodes indicate bootstrap support values; values below 50 are not shown. Major clades are labelled as follows: A, Phocidae; B, Phocinae; C, Monachinae.

No synapomorphies supporting Phocinae were shared across all MPTs in the analysis. Nevertheless, characters 122, entepicondylar foramen in the humerus present (state 0); 135, head of the metapodials keeled with trochleated phalangeal articulations (state 0); and 139, large manus claws (state 0) supported Phocinae in three MPTs, whereas character 13 (P2–P4 and the first molar are double-rooted (state 2)) was recovered as a synapomorphy in six MPTs.

The analysis incorporating molecular constraints recovered 1546 MPTs of 655 steps under the equal weight analysis. The positions of extinct taxa were highly unstable, and relationships among high taxonomic levels were not resolved (see the strict consensus tree in the supplementary material, figure S2). Under implied weight with K = 3, six MPTs were recovered (fit = 66.00), with a RI of 0.606 and a CI of 0.304 (figure 10). As in the unconstrained analysis, implied weight improved, in part, the overall resolution relative to the strict consensus tree obtained under equal weight. Phocidae was recovered as a monophyletic group, with *A. kernensis* as its closer sister taxon. *Kawas* was placed within Phocidae in an unresolved position, forming a polytomy with *A. chagorum*, *D. claytoni, D. emryi* and two clades including the subfamilies Monachinae and Phocinae. ‘*L. proxima*’ was recovered as the sister taxon to all other phocines. Most nodes showed low support values, with some exceptions.

**Figure 10.**
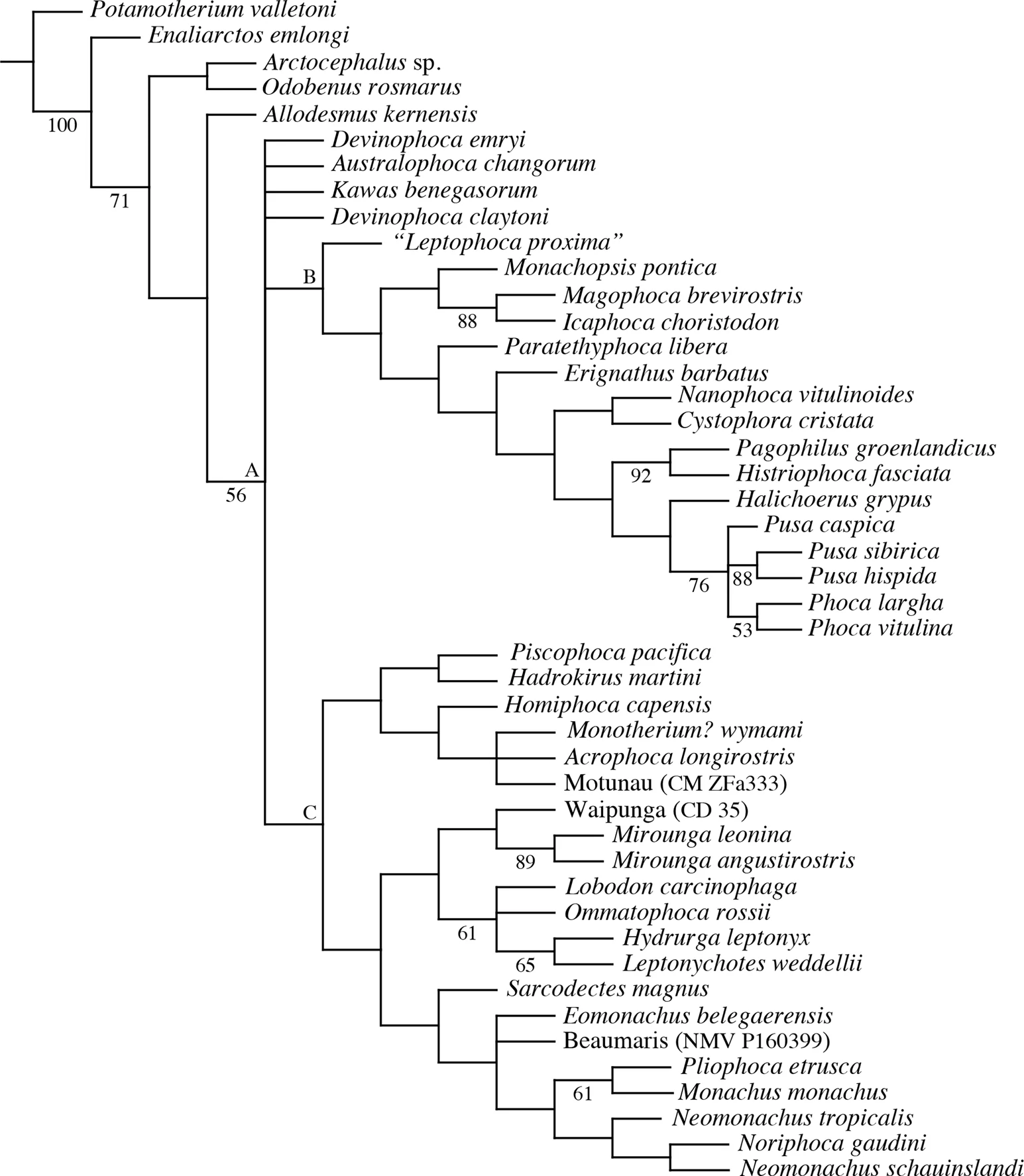
Strict consensus of the six most parsimonious trees resulting from the phylogenetic analysis with implied weighting (K = 3) and a molecular constraint. The numbers below the nodes are bootstrap support values; values below 50 are not shown. Major clades are labelled as follows: A, Phocidae; B, Phocinae; C, Monachinae.

The common synapomorphies shared by Phocidae in the six MPTs are: character 15, absence of M2 (state 1); character 44, strongly or dominantly concave lateral border of opening of nasal cavity in lateral view (state 1); character 52, position of anterior opening of infraorbital foramen in ventral view at the level or posterior to M1 (state 1); character 54, anterior end of jugal in dorsal view above or medial to the lateral margin of the infraorbital foramen (state 1); character 60, post-auricular zygomatic shelf absent (state 0); character 68, lateral end of the tympanic bulla is lateral to level of mid-width of glenoid fossa (state 1); character 71, strong inflation of the tympanic bulla (state 1); character 82, postero-medial extension of the tympanic, past the carotid foramen is absent, bulla contacts and is fused to the exoccipital and mastoid (state 0); character 93, heavily pachyosteosclerotic mastoid present (state 1); character 98, relation of paroccipital process to mastoid where the paraoccipital process is well separated from mastoid (state 1).

## 5. Discussion

### 5.1. Age and affinities within Phocidae

The evolutionary relationships within Phocidae remain partially resolved. Although molecular data provide a robust framework for extant species, the position of extinct seals and the early diversification of the group continue to be debated (e.g. [9,41–43]). Within this context, *K. benegasorum* is particularly informative. This species was originally assigned to Phocinae based on qualitative features of the fore- and hind limbs, making it the only record of an extinct phocine in the Southern Hemisphere [5]. However, subsequent studies have shown that phocid postcranial morphology exhibits substantial intra- and interspecific variation (e.g. [7,10,44]), limiting the diagnostic value of many of these traits at the subfamily level. Our redescription and comparison of the holotype show that traits previously regarded as supporting *Kawas* affinities to Phocinae are not exclusive to that subfamily but are instead variably present among Monachinae.

Several traits emphasized in the original description, including the entepicondylar foramen and a prominent supinator crest of the humerus, are no longer reliable indicators of phocine affinity. Although the entepicondylar foramen has been considered plesiomorphic for Phocinae [33], it also occurs in extinct monachines such as *Homiphoca* and *Magophoca* and varies among extant phocines [7,8,39]. Likewise, although a well-developed supinator crest is typical of most phocine seals [45], its development is variable within Monachinae, from reduced in *Neomonachus* to conspicuous in *Pliophoca* [7,40].

*Kawas* was originally distinguished from members of Monachinae by an everted ilium, a prominent femoral medial epicondylar crest and unreduced pedal ungual phalanges [5]. However, its approximate 62° iliac eversion falls within the monachine range and at the lower limit of phocines [37]. Moreover, its femoral medial epicondylar crest lacks the pronounced distolateral distortion in phocines, instead resembling the state of extinct monachines like *Magophoca* [8]. Finally, the pedal ungual phalange is intermediate between the well-developed condition of phocines and the more reduced state of monachines. Within Monachinae, Cozzuol [5] distinguished *Kawas* from lobodontins based on a developed scapular spine and the short epiphyses on the ungual phalanges of the fore-flipper. Although the scapular spine of *Kawas* is incompletely preserved, its base suggests that it was relatively low, aligning more closely with the condition in *Magophoca*, *Mirounga* and *Neomonachus*. Similarly, the short epiphyses of the fore-flipper ungual phalanges of *Kawas* differ from the elongated state observed in Lobodontini, reinforcing the morphological distinction between *Kawas* and the latter group.

In addition to the postcranial traits discussed above, other characters are informative for assessing the affinities of *Kawas* within Phocidae. The post-canine teeth bear a conical main cusp with mesial and distal accessory cusps that are partially fused to the main cusp (figure 2), a condition common among most extinct monachine seals. As noted above, the scapular spine of *Kawas* is incompletely preserved; however, its inferred position suggests that the supraspinatus fossa occupied nearly half of the scapular surface. This configuration differs from that of Phocinae, in which the supraspinatus area is similar in extent to the insertion areas of the infraspinatus and teres major muscles. The humerus provides additional insights. In *Kawas*, the deltopectoral crest is elongated and extends into the distal third of the shaft, gradually tapering distally, as observed in all extinct and some extant Monachinae. This contrasts with phocine seals, in which the deltopectoral crest is usually restricted to the anterior half of the humerus and often terminates abruptly (figure 5; e.g. [37,46]).

In *Kawas*, the pectoralis inserts distally along the deltopectoral crest, matching the monachine condition and differing from the more proximal insertion of phocines [39]. A flattened ulnar styloid and a three-vertebra sacrum further support morphological affinities with Monachinae. The innominate provides additional clues. The anterodorsal process forms an obtuse, nearly right angle, as in several monachine taxa, rather than the acute angle typical of phocines. Although *Kawas* also exhibits well-developed posterodorsal and posteroventral processes, which are often associated with Phocinae, these are also present in some extinct Monachinae (e.g. *Magophoca*). The gluteal fossa is also relatively deep, intermediate between the shallow condition of most Monachinae and the deeply excavated fossa of Phocinae [39]. The femur would further support monachine affinities, with a prominent lateral epicondyle and a patellar facet that is mediolaterally broader than proximodistally high. *Kawas* possesses a well-developed trochanteric fossa, a feature once thought absent in Monachinae [5], but now recognized in several extant and extinct monachine taxa [7]. Finally, the distal articular surface in phalanges is saddle-shaped, similar to Monachinae, and unlike Phocinae, where the articular surfaces are generally cupped.

These observations indicate that no single postcranial or dental character provides robust support for assigning *K. benegasoum* to Phocinae. Instead, the species displays a mosaic of phocine- and monachine-like traits, with its overall morphology showing stronger affinities with Monachinae. This ambiguous morphological signal is consistent with the inconsistent phylogenetic position of *Kawas* across previous analyses. The species has been recovered as a phocine [5,26], a stem phocine [37,42], a stem phocid [9] and a monachine [8]. The latter result may be influenced by taxon sampling biased towards Monachinae, potentially affecting tree topology.

Our analyses similarly recovered *Kawas* in different positions depending on the analytical framework, such as a phocine under morphological analysis or in an unresolved position within Phocidae when molecular constraints were incorporated. These results suggest that its position is sensitive to analytical parameters, likely because its combination of characters does not provide a strong and consistent signal for its placement within a phocid subclade. More broadly, pinniped phylogenetic analyses that incorporate fossil taxa have often high levels of missing data and a strong bias towards cranial characters. This issue is particularly relevant for *Kawas*, which is known primarily from postcranial remains. These limitations likely reduce the ability of phylogenetic analyses to resolve the relationships of morphologically distinct but incompletely known taxa, contributing to instability in tree topology and to persistent disagreement regarding the relationships among pinniped clades. Until more complete specimens are recovered, the phylogenetic position of *Kawas* is likely to remain controversial and difficult to resolve.

### 5.2. *Kawas* stomach contents and inferred feeding preferences

Fossilized stomach contents are rarely preserved in marine tetrapods, being documented only in a few fossil cetaceans and other taxa (e.g. [1,47–49]). This scarcity limits our ability to reconstruct trophic specializations in fossil marine vertebrates, including early pinnipeds, whose foraging ecology has largely been inferred from indirect proxies such as stable isotope analyses (e.g. [23]), dental microwear (e.g. [50,51]) and functional morphology (e.g. [52–55]). Direct evidence of fossil pinniped prey items is rare, further limiting our understanding of the origin and evolution of ecological specializations within the group. Therefore, specimens preserving prey remains, such as *Kawas*, offer a unique opportunity to reconstruct diet and advance the ecological roles of extinct marine predators.

The abundant bony fish elements associated with *Kawas* are interpreted as preserved stomach contents based on different lines of evidence. Fish remains were recovered from the same stratigraphic horizon as MPEF-PV 601 and in close association with the skeleton, forming a concentrated accumulation within *Kawas*’ thoracoabdominal region. No comparable concentrations of fish remains have been documented elsewhere in the Puerto Madryn Formation (e.g. [14,18]). Furthermore, MPEF-PV 601 and the associated fish remains were found in a low-energy depositional environment, characterized by bioturbated muddy sandstones with grains finer than those of the fish remains (figure 1), further reducing the likelihood of post-depositional transport. Alternative explanations are also unlikely. Postmortem scavenging by fish and the subsequent death of one or more individuals is not supported based on the absence of scavenging marks on *Kawas'* skeleton. Likewise, the fragmentary nature and spatially constrained distribution of the fish remains seem to be inconsistent with the accidental accumulation of ecologically unrelated animals [56]. Instead, the available evidence supports their interpretation as ingested prey within the body cavity.

These remains were identified to correspond to ray-finned fishes assigned to the orders Pleuronectiformes, Perciformes, Scorpaeniformes and Gadiformes, which have been documented in bone-bearing horizons of the Puerto Madryn Formation [14,57,58]. These taxa are predominantly demersal to benthopelagic, inhabiting continental shelf environments. Specifically, pleuronectiforms (flatfishes) primarily occupy soft-bottom shelf habitats [59]; serranids (groupers and sea basses) are associated with rocky reef ecosystems [60–63]; and scorpaenids (rockfish and other bottom-dwelling venomous fish) prefer structurally complex substrates in shallow to mid-shelf settings (e.g. [64]) (supplementary material, table S2). Additionally, gadiformes taxa, including members of Gadidae (cod-like fishes), Moridae (moras) and Merlucciidae (merlucciid hakes), are largely demersal to benthopelagic, occupying continental shelf environments, foraging near the sea-floor [65]. The dominance of demersal and benthic species suggests that *Kawas* was piscivorous, targeting fishes within coastal and inner-shelf benthic habitats. However, the preserved assemblage likely represents only a subset of the original diet, as differential digestion and preservation biases may have limited the representation of other prey types (e.g. soft-bodied cephalopods).

### 5.3. Deep time dietary and ecological convergence in the southwestern South Atlantic

The Peninsula Valdés region and its surrounding coastal areas in northern Patagonia are today a highly productive marine biodiversity hotspot [66,67] and a critical habitat for South American sea lions (*Otaria byronia*, *sensu* [68]), fur seals (*Arctocephalus australis*) and southern elephant seals (*Mirounga leonina*) [69–74]. Despite their spatial overlap, these species exhibit distinct foraging strategies. Although exceptions have been reported, sea lions and male elephant seals are primary coastal and benthic predators, whereas fur seals and female elephant seals are primary pelagic predators, often foraging beyond the continental shelf [75–84]. Among coastal and benthic foragers in the region, sea lions preferentially feed on a diverse assemblage of fish, including Gadiformes (notably Moridae, Merlucciidae and Gadidae), Ophidiiformes, Pleuronectiformes, Perciformes and Scorpaeniformes [85–87], whereas larger body-sized male elephant seals can also incorporate pelagic resources during deeper or offshore diving [88].

The stomach contents of *Kawas* provide evidence of a predominantly piscivorous diet, composed of coastal, demersal and benthic fishes. This trophic signal broadly resembles the foraging preferences of extant South American sea lions and male elephant seals in the region, which exploit continental shelf environments and consume benthic prey, while also often incorporating pelagic species. Although the available evidence supports an association with coastal foraging, it does not exclude the possibility that *Kawas* could have exploited offshore environments or consumed soft-bodied prey of unlikely preservation. In this context, the fossil evidence indicates that the exploitation of benthic and demersal prey was already established among pinnipeds in the Late Miocene in the western South Atlantic (figure 11). The repeated use of these resources by non-closely related pinniped lineages (i.e. *Kawas*, *Mirounga* and *Otaria*) likely reflects dietary convergence associated with long-term ecological stability [89]. The Patagonian continental shelf has sustained diverse fish assemblages since the Neogene, maintaining coastal habitats and prey availability despite significant climatic fluctuations and marine transgression events at local [14] and global [90] scales. Such persistence likely promoted the repeated ecological structuring of marine predator communities, favouring the evolution of pinniped lineages (and other marine vertebrates) adapted to coastal and benthic niches.

**Figure 11.**
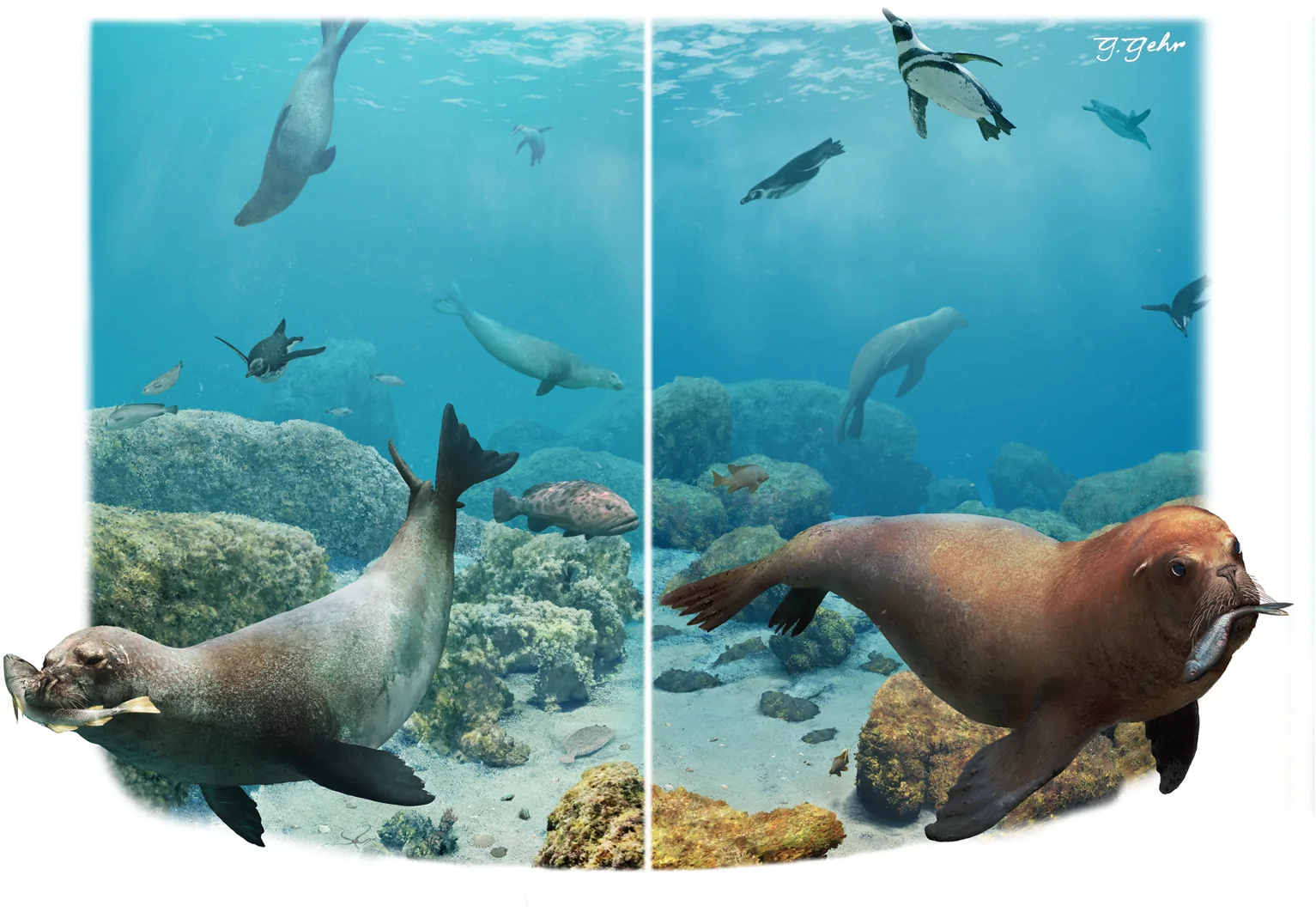
Reconstruction of deep-time dietary convergence. Left, *Kawas benegasorum*; right, the extant South American sea lion, *Otaria byronia*. Both taxa are interpreted as exploiting coastal and benthic food resources. Illustration by Guilherme Gehr.

Body size and functional morphology likely facilitated the local long-term persistence of coastal foraging strategies. The skeleton of *Kawas* belongs to a physically mature seal with an estimated body length of approximately 1.9–2.2 m (based on [31] and [32], respectively), which is within the size range of subadult male (and adult female) South American sea lions [85,91]. Medium- to large-bodied pinnipeds are physiologically well suited for nearshore and shelf foraging, as increased body size generally enhances diving and fasting capacities while allowing for the handling of a broader prey spectrum (e.g. [92–95]). Furthermore, interspecific competition may have reinforced these coastal preferences by favouring niche segregation. Although no additional pinniped taxa have been described from the Puerto Madryn Formation, their occurrence cannot be ruled out as additional, yet unidentified, seal fossil remains have recently been recovered from this unit ([13]; F Busker, M Buono 2025, personal observation). However, morphological comparisons with *Kawas* are currently precluded by the lack of overlapping material. Regardless of the presence of co-occurring pinnipeds, the documented occurrence of sharks, large teleosts and sea-birds from the same stratigraphic levels [14] suggests *Kawas* likely operated within a competitive landscape. This diverse predator guild potentially reflects a complex ancient food web, likely structured by niche partitioning (i.e. benthic nearshore versus pelagic offshore foraging), analogous to the ecological segregation observed in fossil and modern marine communities [94].

## 6. Conclusions

The re-evaluation of the postcranial and dental morphology confirms that *K. benegasorum* possesses a mosaic of features. Although its dentition and most postcranial elements resemble patterns observed in extinct monachine seals, our phylogenetic analysis places *Kawas* either within Phocinae or in an unresolved position within Phocidae, depending on the analysis. The exceptional preservation of stomach contents provides evidence of a predominantly piscivorous diet focused on coastal and benthic prey. These results suggest a case of dietary convergence across geological time, broadly comparable to ecological patterns observed in modern pinniped assemblages of northern Patagonia.

## Supplementary Material



## Data Availability

All new data, including morphological measurements and edits to the previously published morphological matrix used for phylogenetic analyses [9], are provided in the manuscript as a text and as embedded tables and in the supplementary material [96].
