# Supplementary material for: Deep time dietary convergence evidenced by the stomach contents of a fossil seal from the Southwest Atlantic
Source: R Soc Open Sci. 2026 Oct 21;13(10):260705. doi: 10.1098/rsos.260705 (PMC13682869; doi:10.1098/rsos.260705)
Supplement: Supplementary file 1 [file rsos.260705.zip › rsos.260705/rsos260705_si_001.docx]

**Supplementary Material**

**Deep time dietary convergence evidenced by the stomach contents of a fossil seal from the Southwest Atlantic**

Ana M. Valenzuela-Toro^1,2^, Felipe Busker^3^, José Cuitiño^3^, Néstor Garcia^4^, Mónica Buono^3^

^1^Área Científica, Centro de Investigación y Avance de la Paleontología e Historia Natural de Atacama, CIAHN Atacama, Caldera, Región de Atacama, Chile.

^2^Department of Paleobiology, National Museum of Natural History, Smithsonian Institution, Washington DC, USA.

^3^Instituto Patagónico de Geología y Paleontología (IPGP), CCT CONICET CENPAT. Puerto Madryn, Chubut, Argentina.

^4^Centro para el Estudio de Sistemas Marinos (CESIMAR), CCT CONICET CENPAT. Puerto Madryn, Chubut, Argentina.

**Corresponding Author Email:** anavalenzuela@ciahn.cl

**Figure S1.** Strict consensus tree of 7,708 most parsimonious trees resulting from the phylogenetic analysis with equal weighting. The numbers below the nodes are bootstrap support values; values below 50 are not shown.

**Figure S2.** Strict consensus tree of 1,546 most parsimonious trees resulting from the phylogenetic analysis with equal weighting and a molecular constraint. The numbers below the nodes are bootstrap support values; values below 50 are not shown.

**Table S1**. Morphological character coding modified and added for the phylogenetic analysis. Character missing: “?”. Characters based on Rule et al. (2020).

| **Taxa** | **Character coding** |
| --- | --- |
| *Kawas benegasorum* | ?????????????1?100001011??????????????????????????????????????????????????????????????????????????????????????10???00???101?????0010??000101101?1010??0110?????0?0?????? |
| *Paratethyphoca libera* | ???????????????????????????1000???002???00100?0?????????01???????????????????????????????????????????????????010100100000000000011????0????????????????????????????101?? |
| *Icaphoca choristodon* | 1110110001002111010111110121011000000103011101010001010100000001??1100100111000?102?0??????2100?011?1??1?0001??????????????????????????????????????????????????????????? |
| *Monachopsis pontica* | 11?011?00100201?0000000?010?????????2??30011110???0111?1010???1???0???110????0???00?-???????1????1???????10??11100000111000000101021???10??2010?101?010010112??0??0????? |
| *Magophoca brevirostris* | 11101100011020110111111101010101000001030111010100010101000010010?110010010?000010210??????21100011?1??1?1011111001001?10(01)000011012101(01)????10101?01001111011211100001??? |

**Table S2**. Taxonomic list with ecological and biological information of the groups of fishes registered in the stomach contents of *Kawas* *benegasorum*.

| **Classification** | | **Bone element** | **Habitat** | **Size** | **Depth range** | **Reference** |
| --- | --- | --- | --- | --- | --- | --- |
| **Order** | **Family** |  |  |  |  |  |
| Pleuronectiformes | Bothidae | Pelvic girdles and caudal vertebrae with urostyles | Benthic coastal | 50 cm (frequent size) (up to 105 cm) | up to 120 m | 1 |
| Perciformes or Scorpaeniformes |  | Pterygoids, dorsal spines, and preoperculars with serrated margins | Coastal, reef environments, temperate-to-cold waters | up to 40 cm  44-50 cm | up to 130m  up to 300m | 1 |
| Gadiformes | Gadidae or Moridae | Premaxillae with teeth and dentary with teeth | Benthopelagic, temperate waters.  Coastal, reef environment,  cold waters | 15-20 cm  30 - 90cm | 50-900 m.  30-400 m  (usually > 200 m) | 2 |
|  | Merlucciidae | Premaxillae with teeth | Benthopelagic, temperate waters | 60cm (frequent size) | 50-800m (usually 100-200m) | 1 |

**References**

1. <https://www.proyectoarrecife.com.ar/>
2. <https://fishbase.se/>
